# An Evidence-Based Update on the Potential for Malignancy of Oral Lichen Planus and Related Conditions: A Systematic Review and Meta-Analysis

**DOI:** 10.3390/cancers16030608

**Published:** 2024-01-31

**Authors:** Miguel Ángel González-Moles, Pablo Ramos-García

**Affiliations:** 1 School of Dentistry, University of Granada, 18071 Granada, Spain; pramos@correo.ugr.es; 2 Instituto de Investigación Biosanitaria ibs.GRANADA, 18012 Granada, Spain

**Keywords:** oral lichen planus, oral potentially malignant disorder, oral cancer, systematic review, meta-analysis

## Abstract

**Simple Summary:**

Lichen planus (LP) is a chronic inflammatory mucocutaneous disease of autoimmune nature and unknown etiology, which can affect the oral mucosa, skin, nails, scalp, genitalia, and other mucous membranes. The anatomical location most frequently affected by LP is the oral cavity—called oral lichen planus (OLP)—where white reticular lesions may also be accompanied by erosive, atrophic, bullous, papular, or plaque lesions. The most important feature of OLP is its capacity to develop into oral cancer throughout the course of the disease, which is why OLP is currently recognized as an oral potentially malignant disorder (OPMD). New primary-level studies (n = 20; 11,512 patients suffering from OLP or related lesions) have been published in the last 5 years on this topic. In the present meta-analysis, we provide an updated OLP malignant transformation ratio, which is higher than what was previously reported; resolve some remaining controversies; and provide new recommendations for clinical practice in the management of OLP patients.

**Abstract:**

A systematic review and a meta-analysis is presented on published articles on the malignant transformation of oral lichen planus (OLP) and related conditions, which, based on current evidence, updates an earlier systematic review published by our research group that included publications until November 2018. In this updated study (Nov-2023) we searched MEDLINE, Embase, Web of Science, and Scopus. We evaluated the methodological quality of studies (QUIPS tool) and carried out meta-analyses. The inclusion criteria were met by 101 studies (38,083 patients), of which, 20 new primary-level studies (11,512 patients) were published in the last 5 years and were added to our updated study. The pooled malignant transformation ratio was 1.43% (95% CI = 1.09–1.80) for OLP; 1.38% (95% CI = 0.16–3.38) for oral lichenoid lesions; 1.20% (95% CI = 0.00–4.25) for lichenoid reactions; and 5.13% (95% CI = 1.90–9.43) for OLP with dysplasia. No significant differences were found between the OLL or LR groups and the OLP subgroup (*p* = 0.853 and *p* = 0.328, respectively), and the malignant transformation was significantly higher for the OLP with dysplasia group in comparison with the OLP group (*p* = 0.001). The factors that had a significant impact with a higher risk of malignant transformation were the presence of epithelial dysplasia, a higher methodological quality, the consumption of tobacco and alcohol, the location of lesions on the tongue, the presence of atrophic and erosive lesions, and infection by the hepatitis C virus. In conclusion, OLP behaves as an oral potentially malignant disorder (OPMD), whose malignancy ratio is probably underestimated as a consequence essentially of the use of inadequate diagnostic criteria and the low methodological quality of the studies on the subject.

## 1. Introduction

Lichen planus (LP) is a mucocutaneous disease which can affect the oral mucosa, skin, nails, scalp, genitalia, and other mucous membranes [1,2]. It usually presents as a chronic or relapsing disorder of unknown etiology whose pathogenesis involves a T-lymphocyte-mediated autoimmune aggression directed towards the keratinocytes of the epidermis and squamous epithelium of the various mucous membranes [3,4]. The most characteristic clinical feature of LP is the presence of white hyperkeratotic striae [5]. The site most frequently affected by lichen planus is the oral mucosa—called oral lichen planus (OLP)—where white striae may occur as single lesions or are accompanied by erosive, atrophic, bullous, papular, or plaque lesions. The most important feature of OLP is its capacity to develop into oral cancer throughout the course of the disease, which is why OLP is currently considered, after years of controversy [6], to be an oral potentially malignant disorder (OPMD) [7]. In February 2020, a symposium of experts in OPMDs, convened through the WHO Collaborating Centre for Oral Cancer in King’s College London, was held in Glasgow in order to present a revised classification of OPMDs, with nomenclature and definitions for each disorder [7]. In addition, the malignancy ratios of each of the PMDs affecting the oral cavity were also updated and advances were made [7,8,9,10,11,12,13,14,15,16,17], as far as possible, in the resolution of the existing controversies in this regard; all this was performed on the basis of the results obtained through systematic reviews and meta-analyses, therefore providing the best evidence available at that moment. For example, oral submucous fibrosis (OSMF) was defined as “a chronic, insidious disease that affects the oral mucosa, initially resulting in loss of fibroelasticity of the lamina propria and as the disease advances, results in fibrosis of the lamina propria and the submucosa of the oral cavity along with epithelial atrophy” [7] and the corresponding systematic review and meta-analytical study confirmed that 4.2% of patients diagnosed with OSMF may develop oral cancer [12]. It should be taken into consideration that the OPMDs included in the latest classification of the WHO working group [7] are presented in a heterogeneous way compared to the clinical point of view, which is relevant in the case of OLP, as it can lead to diagnostic confusions. It is important that studies of OLP malignancy include their diagnostic criteria, which should be derived from evidence-based expert consensus.

One of the authors of this paper (MAGM) was convened to that consensus meeting to update the concepts, classification, and malignant potential of OLP, which was derived from the author’s research experience in this field, especially from his recent contributions to the knowledge on the malignant transformation capacity of OLP derived from a meta-analysis published in *Oral Oncology* [18] which is having a remarkable international impact (213 citations from 2019 to the present time). In that meta-analysis, it was shown that OLP malignized in 1.14% of cases and that a series of factors increased the risk of malignization of the disease, among which were tongue location, the presence of erosive lesions, tobacco and alcohol consumption, and, above all, the presence of epithelial dysplasia. Likewise, probable factors, such as HCV infection, appeared to increase the risk of malignization, although the lack of robustness of these results was recognized due to the scarcity of primary-level studies on the subject. Our meta-analysis also resolved controversies related to the general acceptance by clinicians and researchers of not enough evidence-based information derived from the works of van der Meij et al. [19], which were spread with a snowball effect, and which considered oral lichenoid lesions (OLLs) as the only responsible factor for the phenomena of malignant transformation and not attributing to OLP any capacity to evolve to cancer. Our study demonstrated on the basis of the best evidence that this was not true and consequently provided crucial information applicable to clinical practice, justifying the need to meticulously examine OLP patients for incipient carcinomas, to clearly inform patients about this aspect, and to follow them throughout their lives. Our research group has reported [20] that the malignancy rate of OLP is probably underestimated, among other reasons, due to the low methodological quality of the primary-level studies on the subject [10] and we have reported that the malignancy rate increases to 2.28% of OLP cases if only primary-level studies performed with the highest methodological quality are included in the meta-analysis.

The ability of OLP to behave as an OPMD and consequently to be able to progress to oral cancer continues to attract great interest among clinicians and researchers in oral medicine and oral pathology, as attested by the publication of 20 new primary-level studies on the topic, providing additional information on 11,512 patients since the final search date used in our 2019 meta-analysis [18]. These new studies have been included in a new meta-analysis, adding up to a total sample of 101 papers analyzing 38,083 patients with OLP and related conditions. In this meta-analysis, we provide an updated OLP malignancy ratio, which is higher than the one that was previously reported; resolve some controversies that were still pending; and provide new recommendations for clinical practice applicable to the management of patients with OLP.

## 2. Materials and Methods

The present systematic review and meta-analysis updates the information published in our previous meta-analysis [18] and complies with the MOOSE guidelines and PRISMA statement [21,22].

### 2.1. Protocol

Earlier, we registered a protocol outlining our methodology aimed at mitigating the risk of bias and enhancing the integrity, precision, and transparency of the ongoing systematic review and meta-analysis. The study protocol has been officially registered with PROSPERO, the international prospective register of systematic reviews under the registration number CRD42019128539 (accessible at www.crd.york.ac.uk/PROSPERO (accessed on 25 December 2023)) [23]. It is worth noting that the protocol diligently adhered to the PRISMA-P reporting guidelines, ensuring a rigorous approach [24].

### 2.2. Search Strategy

In our previous published work, Pubmed, Embase, Web of Science, and Scopus databases were searched for studies published before November 2018 [18]. We updated the bibliographic search, searching for recent studies published in the last five years (upper limit = November 2019; no lower date limit). The search strategy employed a combination of thesaurus terms utilized by the databases (such as MeSH terms) and free terms. To optimize sensitivity, the search strategy integrated the following terms: (“Lichen Planus, Oral” [Mesh] or “oral lichen planus” [All Fields] or “olp” [All Fields] or “oral lichenoid lesion” [All Fields] or “oll” [All Fields]) and (malign* or premalign* or “potentially malignant disorder” or “precancer” or “cancer” [All Fields] or “Carcinoma, Squamous Cell” [Mesh] or “squamous cell carcinoma” [All Fields] or “oscc” [All Fields] or “transformation” [All Fields] or “risk” [All Fields] or “progression” [All Fields]). The full syntax for all databases can be found in the Supplementary Information (Table S1). All potentially included papers were managed using Mendeley v.1.19.8 (Elsevier, Amsterdam, The Netherlands). The duplicate references were removed using this software.

### 2.3. Eligibility Criteria

Eligibility criteria were coincident with those applied in our previous work [18].

Inclusion criteria: (1) Primary-level studies published in English on the potential for malignancy of OLP; (2) longitudinal study design; (3) if findings arise from overlapping populations of patients with OLP, priority was given to the latest reported results or those offering more comprehensive data for inclusion.

Exclusion criteria: (1) Retracted articles, narrative reviews, systematic reviews with or without a meta-analysis, case reports, letters, editorials, meeting abstracts, comments or personal opinions, book chapters, and any non-English language study. (2) Animal experimentation or in vitro studies. (3) Studies not analyzing the OLP malignant transformation risk or providing insufficient data for its calculation. (4) Cross-sectional study design in which OLP patients were not followed up. (5) Studies not differentiating between cutaneous and oral lichen planus (or lichen planus from other anatomical sites).

Two researchers (MAGM and PRG) conducted a two-phase article selection process. In the first phase, the titles and abstracts were screened to identify articles meeting the inclusion criteria. The second phase involved reading the full text of the selected articles and excluding those that did not meet the eligibility criteria.

### 2.4. Data Extraction

The authors (MAGM and PRG) extracted data from the included primary-level studies using Microsoft Excel v.2015 spreadsheets (Microsoft, Redmond, WA, USA). The extracted datasets included information such as the study authors, affiliations, year of publication, country and continent, study design, sample size (number of patients with OLP and number of malignization cases), recruitment period, follow-up information, criteria for OLP diagnosis, clinical appearance and location of OLP lesions, sex and age distribution, tobacco and alcohol consumption, as well as the presence of hepatitis C infection and/or diabetes mellitus.

### 2.5. Evaluation of Quality and Risk of Bias

The authors (MAGM and PRG) critically assessed the methodological quality and risk of bias across primary-level studies using the Quality in Prognosis Studies (QUIPS) tool. This tool, originally developed by scientific members of the Cochrane Prognosis Methods Group, was employed for the comprehensive evaluation of the methodological aspects and potential biases in the included studies [25,26]. The QUIPS tool comprises the following risk of bias domains: Domain 1, study participation; Domain 2, study attrition; Domain 3, prognostic factor measurement; Domain 4, outcome measurement; Domain 5, study confounding; and Domain 6, statistical analysis and reporting [25]. The methodological quality was critically evaluated and scored, with the results visually depicted in a plot for descriptive and clarity purposes. The evaluation considered the risk of bias in each domain as low (assigned 3 points, represented by green color), moderate (assigned 2 points, indicated by yellow), or high (assigned 1 point, depicted in red). Employing this quantitative system, an overall score was also computed for each study. A study achieving a score of ≥14 out of 18 was classified as having high methodological quality, serving as a cutoff point for sensitivity analysis purposes [10].

### 2.6. Statistical Analysis

The malignization ratios for oral lichen planus (OLP), oral lichenoid lesions (OLLs), lichenoid reactions (LRs), and OLP with dysplasia were separately estimated in the meta-analysis by combining their proportions and corresponding 95% confidence intervals (CIs). These proportions were calculated by extracting raw numerators (total number of malignant transformation cases) and denominators (total number of cases). The 95% CIs were estimated for primary-level studies according to the score-test statistic. The influence of studies with extremely small values (i.e., malignant transformation proportion equal to 0 or close) was minimized by using Freeman–Tukey double-arcsine transformation to stabilize the variance of proportions [27]. Pooled proportions were estimated by applying a random-effects model [28]. Furthermore, additional meta-analyses were performed to establish the capacity of study covariates (sex, clinical appearance and location of OLP lesions, tobacco and alcohol consumption, and HCV infection) as potential predictors of the risk of progression to cancer. These meta-analyses were performed by combining relative risks (RR) and their corresponding 95% CIs using both fixed-effect and random-effect (DerSimonian and Laird method) models [29]. Forest plots were developed in order to graphically represent the study results and for subsequent analysis. The χ^2^-based Cochran’s Q test was used to assess the between-study heterogeneity [30]; given its low statistical power, *p* < 0.10 was considered to assume significant heterogeneity. The Higgins I^2^ statistic was also used to quantify the percentage heterogeneity, with results of 25, 50, and 75% indicating, respectively, low, moderate, and high heterogeneity. Subgroup analyses were performed through stratified meta-analyses using diagnostic criteria and risk of bias (RoB) among the primary-level studies in order to identify possible sources of heterogeneity and determine the malignization proportion in these subgroups [31].

Sensitivity analyses were also carried out in order to assess the influence of risk of bias on the malignant transformation rate. For this purpose, the meta-analyses were repeated only including the subset of the primary-level studies conducted with highest methodological quality [10]. Finally, funnel plots were also developed and the Egger regression test (*p*_Egger_ < 0.10) was carried out in order to analyze small-study effects such as publication bias [32,33,34] (Egger et al., 1997; Sterne et al., 2011; Jin et al., 2015). Stata software was used for all statistical analyses (v.16.1, Stata Corp, College Station, TX, USA).

## 3. Results

### 3.1. Results of the Literature Search

**Figure 1:**
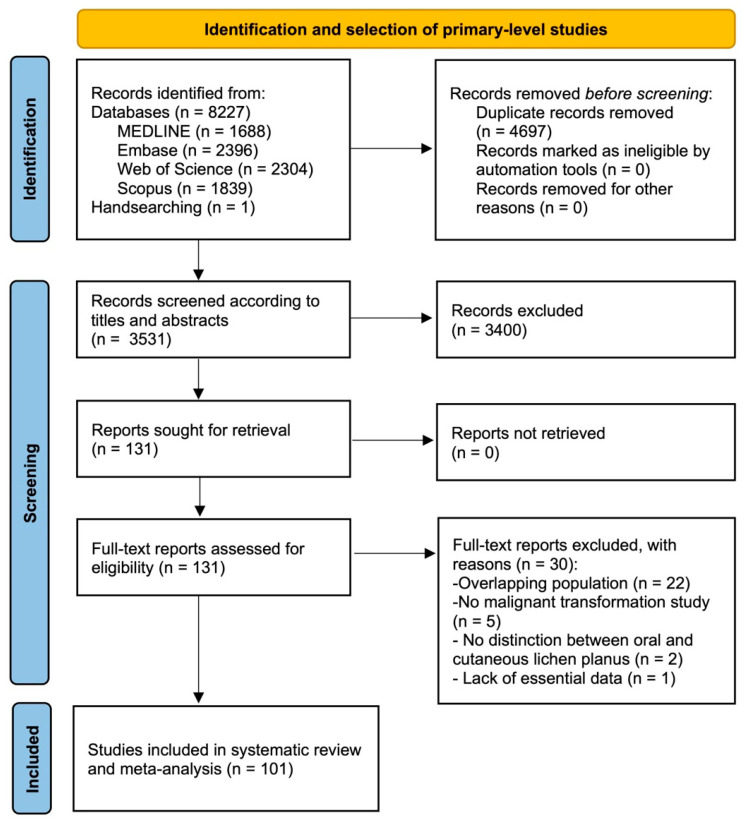
Flow diagram showing the identification and selection process for studies.

The flow diagram depicted in Figure 1 graphically represents the process of identification, screening, and selection of primary-level studies. A total of 8227 records were obtained and were distributed as follows: 2396 from Embase, 2304 from Web of Science, 1839 from Scopus, 1688 from MEDLINE, and 1 from manual searching methods. Following the removal of duplicates, a total of 3521 records underwent screening based on titles and abstracts, resulting in a subset of 131 papers for full-text evaluation. Finally, 101 studies met the eligibility criteria and were included for the qualitative analysis and meta-analysis [20,35,36,37,38,39,40,41,42,43,44,45,46,47,48,49,50,51,52,53,54,55,56,57,58,59,60,61,62,63,64,65,66,67,68,69,70,71,72,73,74,75,76,77,78,79,80,81,82,83,84,85,86,87,88,89,90,91,92,93,94,95,96,97,98,99,100,101,102,103,104,105,106,107,108,109,110,111,112,113,114,115,116,117,118,119,120,121,122,123,124,125,126,127,128,129,130,131,132,133].

### 3.2. Study Characteristics

Table 1 provides a summary of the key characteristics of the incorporated studies, while Table S2 (Supplementary file, pp. 4–7) details the characteristics and variables collected in each study. The study included 101 studies encompassing a total of 38,083 patients, comprising 36,889 with OLP, 856 with OLLs, 164 with LRs, and 174 with OLP with dysplasia. Among these patients, 606 developed oral cancer.

**Table 1:** Summarized characteristics of the study sample. Table S2 (Supplementary file, pp. 4–7) summarizes the characteristics of each study.

Summarized Characteristics of Reviewed Studies	
Total	101 studies
Year of publication	1929–2023
Number of patients	
Total	38,083
Developed oral cancer	606
Sample size, range	16–3568 patients
Diagnostic entity	
Oral lichen planus (OLP)	97 studies (36,889 patients)
Oral lichenoid lesions (OLLs)	8 studies (856 patients)
Lichenoid reactions (LRs)	4 studies (164 patients)
OLP with dysplasia	5 studies (174 patients)
Study design	
Retrospective longitudinal	92 studies
Prospective longitudinal	8 studies
Ambispective longitudinal	1 study
Geographical region	
Europe	51 studies (18 countries)
Asia	26 studies (11 countries)
North America	15 studies (2 countries)
South America	4 studies (2 countries)
Oceania	3 studies (2 countries)
Africa	2 studies (1 country)
Total	6 continents, 36 countries

### 3.3. Qualitative Evaluation

**Figure 2:**
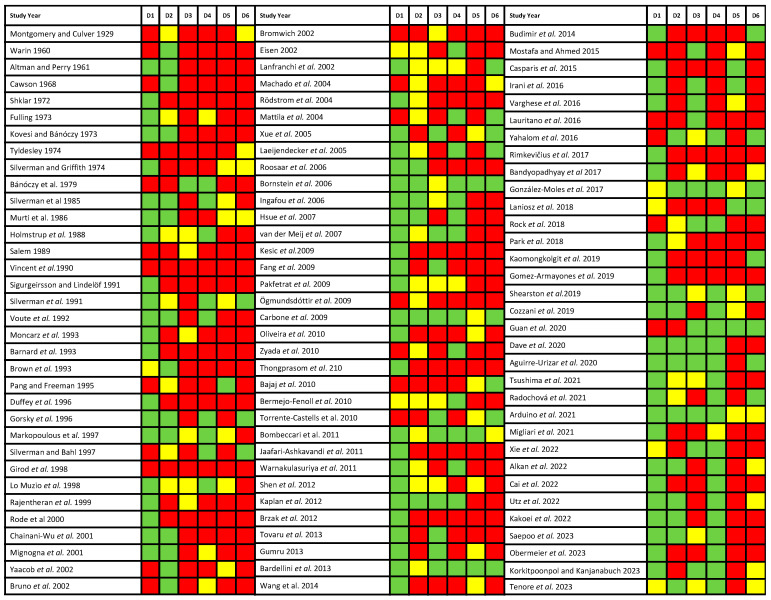
Evaluation of the risk of bias using the Quality in Prognosis Studies (QUIPS) tool. The risk of bias across domains was graphically represented in green color (low risk of bias), yellow (moderate risk of bias) or red (high risk of bias).

In the risk of bias critical appraisal using the QUIPS tool, it can be deduced from Figure 2 that not all studies were conducted with uniform methodological quality. The domains with elevated risk of bias were specifically identified as domain No. 5 (pertaining to study confounding) and domain No. 6 (related to statistical analysis and reporting). In addition, 11 studies were considered within the subset with the highest methodological quality, which were subsequently analyzed for sensitivity analysis purposes.

### 3.4. Quantitative Evaluation (Meta-Analysis)

**Figure 3:**
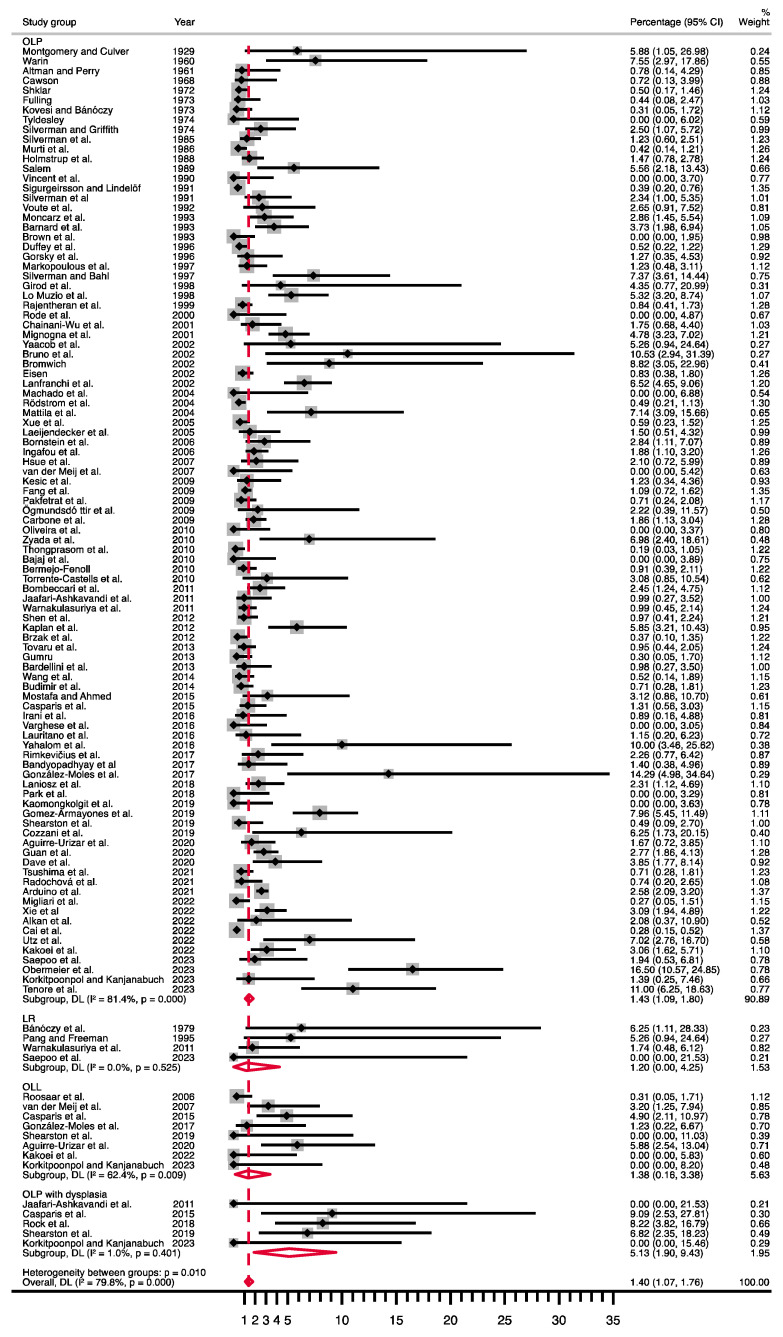
Forest plot graphically representing the meta-analysis on the potential for malignancy of OLP and related conditions. The malignant transformation proportions (expressed as percentages) were stratified by studies that included OLP, OLLs, LRs, and OLP with epithelial dysplasia. Diamonds, depicted in dark red color, indicate the overall and subgroup effect sizes—pooled proportions were used as effect size metric, expressed as percentages—jointly with their corresponding 95% confidence intervals (CIs).

The malignant transformation ratio for the OLP group was 1.43% (95% CI = 1.09–1.80); 1.38% (95% CI = 0.16–3.38) for the OLL group; 1.20% (95% CI = 0.00–4.25) for the LR group; and 5.13% (95% CI = 1.90–9.43) for the OLP with dysplasia group. No significant differences were found between the OLL or LR groups and the OLP subgroup (*p* = 0.853 and *p* = 0.328, respectively), and the malignant transformation was significantly higher for the OLP with dysplasia group in comparison with the OLP group (*p* = 0.001) (Figure 3, Table 2, Supplementary Information).

Furthermore, the risk of oral cancer development was also analyzed for additional secondary variables, and significant statistical differences were found for the diagnostic criteria applying clinical and histopathological parameters (PP = 1.92%, 95% CI = 1.48–2.41, *p* < 0.001), smokers (RR = 1.60, 95% CI = 1.07–2.41, *p* = 0.02), HCV-positive patients (RR = 3.67, 95% CI = 1.48–9.14, *p* = 0.005), OLP lesions located in the tongue (RR = 1.82, 95% CI = 1.25–2.63, *p* = 0.002), and with red clinical aspect (RR = 2.38, 95% CI = 1.85–3.07, *p* < 0.001).

In the sensitivity analysis, the subsets of studies with the highest methodological quality showed higher pooled malignant transformation ratios for OLP (PP = 2.25%, 95% CI = 1.65–2.94) and for OLLs (PP = 2.11%, 95% CI = 0.01–6.33), with no significant differences among both groups (*p* = 0.849). Both the visual inspection analysis of the funnel plot asymmetry and the accompanying statistical test (*p*_Egger_ < 0.001) confirmed the existence of significant small-study effects, as detailed in the Supplementary Information. Consequently, it is not possible to dismiss the presence of publication bias.

**Table 2:** Malignant transformation risk and related variables.

				Pooled Data		Heterogeneity		
Analysis	No. of Studies	No. of Patients	Stat. Model	ES (95% CI)	*p*-Value	*Q*	*P_het_*	*I*^2^ (%)
Diagnosis ^a^					0.001 ^b^			
OLP	97	36,889	R, d-l	PP = 1.43% (1.09–1.80)		515.65	<0.001	81.4
OLP with dysplasia	5	174	R, d-l	PP = 5.13% (1.90–9.43)		4.04	0.40	1.0
Diagnosis ^a^					0.853 ^b^			
OLP	97	36,889	R, d-l	PP = 1.43% (1.09–1.80)		515.65	<0.001	81.4
OLL	8	856	R, d-l	PP = 1.38% (0.16–3.38)		18.62	0.009	62.4
Diagnosis ^a^					0.328 ^b^			
OLP	97	36,889	R, d-l	PP = 1.43% (1.09–1.80)		515.65	<0.001	81.4
LR	4	164	R, d-l	PP = 1.20% (0.00–4.25)		2.24	0.53	0.0
Criteria ^a^					<0.001 ^b^			
Clinical and histopathological	70	27,975	R, d-l	PP = 1.92% (1.48–2.41)		378.36	<0.001	81.8
Clinical or non-exhaustive	31	10,108	R, d-l	PP = 0.61% (0.25–1.07)		110.66	<0.001	72.9
Sex ^c^								
Male vs. Female	59	29,297	F, m-h	RR = 1.13 (0.93–1.38)	0.208	44.81	0.898	0.0
Smoking ^c^								
Smokers vs. non-smokers	24	7122	F, m-h	RR = 1.60 (1.07–2.41)	0.022	20.04	0.581	0.0
Alcohol ^c^								
Drinkers vs. non-drinkers	11	3275	F, m-h	RR = 2.11 (1.13–3.97)	0.020	10.73	0.379	6.8
HCV ^c^								
HCV-positive vs. negative	8	5433	R, d-l	RR = 3.67 (1.48–9.14)	0.005	17.40	0.015	59.8
Localization ^c^								
Tongue vs. others	22	15,284	F, m-h	RR = 1.82 (1.25–2.63)	0.002	11.68	0.948	0.0
Clinical aspect ^c^								
Red vs. white	39	14,515	F, m-h	RR = 2.38 (1.85–3.07)	<0.001	27.90	0.885	0.0
Appraisal of highest quality studies ^d^					0.849 ^b^			
OLP	11	6379	R, d-l	PP = 2.25% (1.65–2.94)		17.49	0.064	42.8
OLLs	3	197	R, d-l	PP = 2.11% (0.01–6.33)		3.69	0.158	45.8

Abbreviations: Stat., statistical; F, fixed-effects model; R, random-effects model; m-h, Mantel–Haenszel method; d-l, DerSimonian and Laird method; ES, effect size estimation; CI, confidence interval; OLP, oral lichen planus; OLL, oral lichenoid lesion; LR, lichenoid reaction; PP, pooled proportion; RR, relative risk. a—proportion meta-analyses (subgroup analyses); b—test for between-subgroup differences; c—prognosis meta-analyses; d—sensitivity analysis.

## 4. Discussion

Our current meta-analysis of malignant transformation of OLP and related lesions was performed on a total of 101 papers providing information on 38,083 patients. Of these papers, 97 primary-level studies (36,889 patients) specifically focused on investigating the malignancy rate of OLP; the meta-analysis found that 1.43% of OLP cases developed oral cancer, a result that derives from the robust evidence, as demonstrated by the narrow confidence interval provided by the analysis (1.03–1.74). We should point out that there is growing interest in the study of the malignancy risk of OLP among clinicians and researchers, as evidenced by the exponential increase in the number of publications on the subject that have appeared in the literature in recent years; thus, between the years 1929 and 2018, the literature search period of our initial meta-analysis published in *Oral Oncology* [18], 78 studies (25,848 patients) were published, while in the 5 subsequent years (November 2018 to November 2023), 20 additional papers (11,512 patients) were published which was analyzed in our meta-analysis and which have reinforced and reaffirmed our knowledge on this important topic that, until very few years ago, was fraught with controversy [6]. In this increased research interest, the conclusions of the expert symposium held in Glasgow in 2020 on OPMDs have probably played a major role, among which, the reported information on OLP malignancy through three papers from our group are having notable international repercussions [7,10,11]. The OLP malignization rate reported in our current meta-analysis (1.43%; 95% CI = 1.09–1.80) is higher than that reported by us in 2019 (1.14%; 95% CI = 0.84–1.49) and that reported by the four meta-analyses published to date on the subject [134,135,136,137], which underlines that the increase in research—and in the sample—makes it possible to obtain a result that is closer to reality.

Our current results, however, reaffirm our previous hypothesis that the malignancy rate of OLP is underestimated [6,10,11,18,20]. In our opinion, this is essentially due to the application of inappropriate diagnostic criteria that exclude cases of OLP with epithelial dysplasia [19,138]. Knowing that the presence of epithelial dysplasia behaves as the main risk factor for the malignant transformation of OPMDs, logic dictates that the exclusion of OLPs with dysplasia from the case series analyzed in studies of OLP malignancy necessarily underestimates the risk of malignancy of this disease. Our present results reconfirm this hypothesis: the malignancy rate of OLP with epithelial dysplasia was significantly higher (5.13%) than that presented by case series that do not provide information on the presence or absence of epithelial dysplasia (*p* = 0.001). We have previously pointed out [139] that the evaluation of epithelial dysplasia in OLP is complex mainly because of the resemblance of the key histological facts of OLP (vacuolizing degeneration of the basal layer of the epithelium) with some features of epithelial dysplasia, which can be especially marked in the case of the diagnosis of mild and moderate dysplasias in which the histopathological alterations are located in the lower layers of the epithelium. However, this fact only points out that the evaluation of dysplasia in OLP should be performed by experienced pathologists and in our view, in no way should the presence of epithelial dysplasia be considered as a diagnostic exclusion criterion for OLP, taking into consideration that there is no evidence to suggest that OLP cannot develop epithelial dysplasia in the course of its evolution to cancer, as occurs in the rest of the OPMDs. We conclude that considering epithelial dysplasia as an exclusion criterion for the diagnosis of OLP should not be applied because it contributes to underestimating the risk of OLP malignancy and because it is not evidence-based. It should also be noted that this is not a trivial issue since of the 97 papers included in our current meta-analysis, 26 used OLP diagnostic criteria that consider the presence of dysplasia as a reason for diagnostic exclusion of the disease. The malignancy rate of OLP is also underestimated due to the low methodological quality of the papers published on the subject. We had previously demonstrated [10] that a meta-analysis restricted to the studies with the highest methodological quality yielded significantly higher OLP malignancy rates than those of the total sample. We have now again selected the 11 studies with the highest methodological quality as measured by the QUIPS tool, through which, we were able to assign a numerical score to all our studies, selecting those that were in the top 25% of the methodological quality score. The meta-analysis of OLP malignancy in these 11 studies yielded a transformation rate of 2.25% (95% CI = 1.65–2.94), notably higher than that found in the overall meta-analysis (1.43%). Our recommendation for the future is that studies on the malignant transformation of OLP should be conducted with high methodological quality, which would imply providing adequate information on sample source, using large sample sizes, correctly documenting the demographic and clinicopathologic characteristics of OLP lesions and carcinomas arising from them, reporting follow-up periods and dropout rates, clearly reporting the diagnostic criteria for OLP used, clearly differentiating OLP, LR, and OLL cases, and performing an adequate statistical analysis.

A very relevant aspect of our research concerns the results obtained regarding the so-called OLLs, a concept coined by van der Meij et al. [19] to refer to those lesions that did not strictly meet their diagnostic criteria (bilateral and symmetric reticular lesions, which may or may not be accompanied by erosive and/or atrophic lesions; presence of vacuolizing degeneration of the basal layer of the epithelium; presence of band-like inflammatory infiltrate in the superficial chorion; and absence of dysplasia). In their case series [19,84], the authors attributed a risk of malignancy exclusively to OLLs, while they consider OLP to not behave like an OPMD. Our meta-analysis and others [18,134,135,136,137] have shown that this is not true. We found that OLLs malignize in 1.38% of cases (95% CI = 0.16–3.38), a rate very similar to that found for OLP (1.43%), which furthermore is not statistically different (*p* = 0.853). The use of the diagnostic criteria for OLP proposed by van der Meij et al. [19] not only underestimates the risk of OLP malignancy but also gives clinicians the false impression that oral lichen planus does not behave like an OPMD, with the serious risk of relaxing the follow-up protocols that must be applied to these patients. At the same line, it should be emphasized that the optimal conditions to improve our ability to diagnose cancer in OLP are obtained when the diagnosis is based on clinical criteria combined with histopathological information of the biopsied tissue. We have found that the malignancy rate reported by studies that diagnose OLP based on clinical findings and histopathology (1.92%, 95% CI = 1.48–2.41) is significantly higher than that reported by those that only base the diagnosis on a clinical analysis (0.61%, 95% CI = 0.25–1. 07), which is probably due to the fact that a clinical analysis alone does not allow us to differentiate with certainty the nature of the red areas that frequently appear in atrophic–erosive OLP; taking into account that the most common clinical sign of early oral carcinoma is the red area [140,141], we must admit that some incipient carcinomas on oral lichen planus manifesting as red areas could be confused with atrophic forms of lichen if they are not biopsied and therefore, it is imperative to perform biopsies in patients with OLP.

Finally, our meta-analysis confirms that there are some factors that increase the risk of developing cancer in OLP. Among them are smoking (RR = 1.60, *p* = 0.022), alcohol consumption (RR = 2.11, *p* = 0.020), HCV infection (RR = 3.67, *p* = 0.005), lingual location of lesions (RR = 1.82, *p* = 0.002), and presence of red lesions (RR = 2.38, *p* < 0.001). The influence of OLP on the development of cancer transcends oral oncogenesis; in this regard, our group published a systematic review and meta-analysis demonstrating the evidence-based association of OLP with premalignant liver disease and with hepatocarcinoma [142].

It is highly likely that the OLP malignancy is directly dependent on the inflammatory environment generated in the autoimmune process [143]. Understanding the oncogenic mechanisms linked to autoimmunity is complex, partly as a consequence of the inherent heterogeneity in the development of the immune response. To date, more than 30 primary-level studies have been published analyzing the influence of the immune response on the malignant transformation of OLP, which frequently report the role of hyperproliferative stimuli and the hyperactivity of oncogenes with tumor suppressor gene responses [144,145]. Nevertheless, there are no systematic reviews or meta-analyses based on evidence regarding this particular subject.

The potential study limitations of the present study mirror those of a previously published meta-analysis [18]. Firstly, the inclusion of studies limited to the English language may result in a potential loss of information published in other languages. To address this concern, we conducted a pilot search examining articles in other languages, exploring the CNKI and LILACS databases as recommended [146,147]. We identified a few potential articles in other languages, but none were suitable for inclusion. Secondly, a significant degree of heterogeneity was observed in the overall OLP malignization proportion. To address this, a random-effects statistical model was applied in the all-proportion meta-analyses, and secondary stratified analyses were performed to obtain more homogeneous subgroups of studies. Lastly, both visual and statistical analyses confirmed the presence of small-study effects, indicating that publication bias-the tendency in health science literature to publish only positive results [148]- cannot be ruled out. Despite the above limitations, our meta-analyses confirms the robust results, demonstrating strong statistical associations between OLP and malignant transformation with several study variables.

## 5. Conclusions

In conclusion, OLP behaves as an OPMD, whose malignancy ratio is probably underestimated as a consequence, essentially, of the use of inadequate diagnostic criteria and the low methodological quality of the studies on the subject. In addition, there are factors that increase the risk of developing cancer in these patients, who should be informed of the need to stop smoking and alcohol consumption. Clinicians should be aware of the importance of performing biopsies and follow-up of their OLP cases, although so far, there is no evidence as to what is the best follow-up program to improve our capacity to make an early diagnosis of oral cancer in these patients. The development of future studies on OLP malignancy should respect the principles for its development based on the highest methodological quality [10].

## Data Availability

The data that supports the findings of this study are available in the Supplementary Material of this article.

## Supplementary Materials

The following supporting information can be downloaded at: https://www.mdpi.com/article/10.3390/cancers16030608/s1, Table S1. Search strategy for each database, number of results, and execution date; Table S2. Characteristics of the study sample; Figure S1. Forest plot graphically representing the meta-analysis of the malignant transformation proportion (expressed as percentage) stratified by studies that only include patients with oral lichen planus with epithelial dysplasia, and oral lichen planus (excluding or taking no account of the presence of epithelial dysplasia). Pooled proportions and 95% confidence intervals (CI) were used as effect size metric; Figure S2. Forest plot graphically representing the meta-analysis of the malignant transformation proportion (expressed as percentage) stratified by oral lichen planus and oral lichenoid lesions. Pooled proportions and 95% confidence intervals (CI) were used as effect size metric; Figure S3. Forest plot graphically representing the meta-analysis of the malignant transformation proportion (expressed as percentage) stratified by oral lichen planus and lichenoid reactions. Pooled proportions and 95% confidence intervals (CI) were used as effect size metric; Figure S4. Forest plot graphically representing the meta-analysis of the malignant transformation proportion (expressed as percentage) stratified by the presence or absence of exhaustive clinical and histopathological diagnostic criteria. Pooled proportions and 95% confidence intervals (CI) were used as effect size metric; Figure S5. Forest plot graphically representing the prognostic meta-analysis of the risk of oral cancer development in males *versus* females with OLP. Relative risk (RR) and 95% confidence intervals (CI) were used as effect size metric; Figure S6. Forest plot graphically representing the prognostic meta-analysis of the risk of oral cancer development in red OLP (vs. white). Relative risk (RR) and 95% confidence intervals (CI) were used as effect size metric; Figure S7. Forest plot graphically representing the prognostic meta-analysis of the risk of oral cancer development in smokers patients with OLP (vs. non-smokers). Relative risk (RR) and 95% confidence intervals (CI) were used as effect size metric; Figure S8. Forest plot graphically representing the prognostic meta-analysis of the risk of oral cancer development in drinkers patients with OLP (vs. non-drinkers). Relative risk (RR) and 95% confidence intervals (CI) were used as effect size metric; Figure S9. Forest plot graphically representing the prognostic meta-analysis of the risk of oral cancer development in HCV-positive patients with OLP (vs. HCV-negative patients with OLP). Relative risk (RR) and 95% confidence intervals (CI) were used as effect size metric; Figure S10. Forest plot graphically representing the prognostic meta-analysis of the risk of oral cancer development in OLP lesions localized on the tongue in comparison to other sites in the oral cavity. Relative risk (RR) and 95% confidence intervals (CI) were used as effect size metric; Figure S11. Forest plot graphically representing the sensitivity analysis on methodological quality. The subsets of primary-level studies with the highest methodological quality were included in this meta-analysis of the malignant transformation proportion (expressed as percentage) stratified by oral lichen planus and oral lichenoid lesions. Pooled proportions and 95% confidence intervals (CI) were used as effect size metric; Figure S12. A funnel plot of estimated transformed proportions against their standard errors, graphically representing the analysis of “small-study” effects on the oral lichen planus malignant transformation ratio.
