# Supplementary material for: An Evidence-Based Update on the Potential for Malignancy of Oral Lichen Planus and Related Conditions: A Systematic Review and Meta-Analysis
Source: Cancers (Basel). 2024 Jan 31;16(3):608. doi: 10.3390/cancers16030608 (PMC10854587; doi:10.3390/cancers16030608)
Supplement: Supplementary file 1 [file cancers-16-00608-s001.zip › cancers-2818990-supplementary.pdf]

Appendix to the manuscript

An evidence-based update on the potential for malignancy of oral lichen planus and related conditions: a systematic review and meta-analysis.

Miguel Ángel González-Moles [a,b]

Pablo Ramos-García [a,b]

a - School of Dentistry, University of Granada, Granada, Spain.

b - Biohealth Research Institute (IBS), Granada, Spain.

Table of contents

1. Search strategy....3

2. Characteristics of analyzed studies....4

3. Meta-analyses on malignant transformation proportions....8

   3.1 Stratified by OLP vs. OLP with dysplasia....8

   3.2 Stratified by OLP vs. OLL....9

   3.3 Stratified by OLP vs. LR....10

   3.4 Stratified by diagnostic criteria....11

4. Sex and malignant transformation....12

5. Clinical appearance of lesions and malignant transformation....13

6. Tobacco and malignant transformation....14

7. Alcohol and malignant transformation....15

8. Hepatitis C virus and malignant transformation....16

9. OLP localization and malignant transformation....17

10. Sensitivity analysis on methodological quality....18

11. Analysis of small-study effects....19

1. Search strategy

Table S1. Search strategy for each database, number of results, and execution date.

Database	Query	Results	Upper date limit
MEDLINE (PubMed)	("Lichen Planus, Oral"[Mesh] or "oral lichen planus"[All Fields] or "olp"[All Fields] or "oral lichenoid lesion"[All Fields] or "oll"[All Fields]) and (malign* or premalign* or “potentially malignant disorder” or “precancer” or “cancer"[All Fields] or "Carcinoma, Squamous Cell"[Mesh] or "squamous cell carcinoma"[All Fields] or "oscc"[All Fields] or “transformation” [All Fields] or "risk"[All Fields] or "progression"[All Fields])	1,688	November 2023
Embase	('oral lichen planus'/exp OR 'oral lichen planus' OR 'olp' OR 'oral lichenoid lesion' OR 'oll') AND (‘malign*’ OR ‘premalign*’ OR 'potentially malignant disorder' OR 'precancer'/exp OR 'precancer' OR 'cancer'/exp OR 'cancer' OR 'squamous cell carcinoma'/exp OR 'squamous cell carcinoma' OR 'oscc' OR 'transformation'/exp OR 'transformation' OR 'risk'/exp OR 'risk' OR 'progression')	2,396	November 2023
Web of Science	TS=(oral lichen planus OR olp OR oral lichenoid lesion OR oll) AND TS=(malign* or premalign* or potentially malignant disorder or precancer or cancer or squamous cell carcinoma or oscc or transformation or risk or progression)	2,304	November 2023
Scopus	TITLE-ABS-KEY(("oral lichen planus" OR "olp" OR "oral lichenoid lesion" OR "oll") AND ("malign*" OR "premalign*" OR "potentially malignant disorder" OR "precancer" OR "cancer" OR "squamous cell carcinoma" OR "oscc" OR "transformation" OR "risk" OR "progression"))	1,839	November 2023
Total			8227

Table S2. Characteristics of the study sample

Study	Year	Country	Hospital/ affiliation	Design (R/P)	Recruitment period	Follow-up			OLP Sample size	OSCC development	Malignant transformation		Site lesions n (M)	OLP/OLL clinical lesions									Diagnostic criteria						Risk markers		Risk factors					Multiple tumor development
						Range months	Mean Median (months)	Frequency (visits per year)			Ratio (%)	Mean time (months)		Red n (M)	White n (M)	Mixed n (M)	Reticular n (M)	Erosive n (M)	Atrophic n (M)	Bullous n (M)	Plaque n (M)	Other n (M)	Oral medicine/ pathology specialist	Criteria used for diagnosis of OLP/OLL	Differentiation or exclusion between OLP/OLL	Clinic and/without histopathologic criteria	Dysplasia Exclusion (Yes/No)	Tobacco Exclusion (Yes/No)	Sex n (M)	Age n (M)	Smoking n +/- (M +/-)	Alcohol n +/- (M +/-)	HCV n +/- (M +/-)	DM n +/- (M +/-)	Diet	
Montgomery and Culver	1929	UK	University of California Medical School, Department of Dermatology	R	1906-1927	≤252	N/A	N/A	17	1	5.88	19	Buccal mucosa>tongue(1)> lips>gingiva	N/A	8	6 (1)	N/A	N/A	N/A	N/A	N/A	Unknown 3	No	Non described	No	Clinic	No	No	F:7(1) M:10	Mean 39.47 years, range 22-67 (34)	N/A	N/A	N/A	N/A	N/A	0
Warin	1960	UK	United Bristol Hospitals (Uk)	R	1949-1959	≤156	N/A	N/A	53	4	7.55	72	N/A (buccal mucosa 2, tongue 1, lip 1)	N/A	N/A	N/A (1)	N/A	N/A (2)	N/A	N/A	N/A (1)	N/A	No	Described	No	Clinic and/without histopathology	No	No	N/A (F:1 M:3)	N/A (28-58-60-68)	N/A (+1)	N/A	N/A	N/A	N/A	0
Altman and Perry	1961	USA	Mayo Clinic (Rochester)	R	1949-1954	72-112	N/A	N/A	128	1	0.78	48	N/A (buccal mucosa 1)	N/A	N/A	N/A	N/A	N/A	N/A	N/A	N/A (1)	N/A	No	Non described	No	Clinic (and histopathology in 30% of cases)	No	No	N/A (F:1)	N/A (56)	N/A	N/A	N/A	N/A	N/A	0
Cawson	1968	UK	Guy's Hospital Medical School (London)	R	N/A	≤112	N/A	N/A	138	1	0.72	N/A	N/A	N/A	N/A	N/A	N/A	N/A	N/A	N/A	N/A	N/A	Yes	Described	No	Clinic and/without histopathology	No	No	N/A	N/A	N/A	N/A	N/A	N/A	N/A	0
Shklar	1972	USA	Harvard School of Dental Medicine	R	N/A	≤180	N/A	N/A	600	3	0.5	N/A	N/A	N/A	N/A	N/A	N/A	N/A	N/A	N/A	N/A	N/A	Yes	Described	No	Clinic and/without histopathology	No	No	N/A	N/A	N/A	N/A	N/A	N/A	N/A	0
Fulling	1973	Denmark	Copenhagen University Hospital	R	1958-1969	1-144	Mean 43.2 Median 34.8	Annually examination	225	1	0.44	30	N/A (tongue 1)	N/A	N/A	N/A	N/A	N/A	N/A	N/A	N/A	N/A	Yes	Non described	No	Clinic and histopathology	No	No	F:151(1) M:74	N/A (52)	N/A (+1)	N/A	N/A	N/A	N/A	0
Kovesi and Bánóczy	1973	Hungary	Semmelweiss Medical University, Budapest.	R	1960-1969	12-112	Mean 36	N/A	326	1	0.36	N/A	N/A (tongue 1)	N/A	N/A	N/A	168	90 (1)	16	27	N/A	papular 1	Yes	Described	No	Clinic and/without histopathology	No	No	F:204(1) M:122	Range 21-80 years (an elderly woman)	+48 (N/A)	N/A	N/A	N/A	N/A	0
Tyldesley	1974	UK	University of Liverpool	R	N/A	≤62	N/A	N/A	60	0	0	0	Buccal and labial mucosa 54>tongue 16>gingiva 14>palate 4 (0)	N/A	N/A	N/A	N/A	N/A	N/A	N/A	N/A	N/A	No	Non described	No	Clinic and/without histopathology	No	No	F:42 M:18 (0)	30-39 8, 40-49 17, 50-59 13, 60-69 15, >69 6 (0)	N/A	N/A	N/A	N/A	N/A	0
Silverman and Griffith	1974	USA	University of California School of Dentistry	R	1955-1973	≤112	Mean 36	N/A	200	5	2.50	N/A	Buccal mucosa 170, gingiva 89, tongue 81, palate 36, flool of mouth 21, lip 2 (N/A)	N/A	N/A	N/A	68	119 (4)	N/A	N/A	13 (1)	N/A	Yes	Described	No	Clinic and histopathology	No	No	F:129 M:71	Mean 52 years	+66 (+2) -134 (-3)	N/A	N/A	N/A	N/A	0
Bánóczy *et al.*	1979	Hungary and Denmark	Semmelweiss Medical University, Budapest and Copenhagen University Hospital	R	N/A	≤240	N/A	N/A	16 (11 Danish, 5 Hungarian) LRs	1 (Hungarian)	6.25	N/A	N/A	N/A	N/A	N/A	N/A	N/A	N/A	N/A	N/A	N/A	Yes	Described	Yes (exclusion)	Clinic and histopathology	No	No	N/A	N/A	N/A	N/A	N/A	N/A	N/A	0
Silverman *et al.*	1985	USA	University of California School of Dentistry	P	N/A	6-120	Mean 67.2	N/A	570	7	1.23	40.32	Buccal mucosa 87(3), gingiva 60(1), tongue 45(2), palate 16, lips 14(1), floor of mouth 8	N/A	N/A	N/A	181(2)	264 (4)	125 (1)	N/A	N/A	N/A	Yes	Non described	No	Clinic and histopathology	No	No	F:384(4) M:186(3)	Mean 52 years, range 16-86 (46-56-60-64-69-70-74)	+27 (+2) -543 (-5)	N/A	N/A	+33 (N/A)	N/A	0
Murti *et al.*	1986	India	Kerala (house to house studies)	R	1967-1977	12-112	Mean 61.2	N/A	722	3	0.41	91.2	Buccal mucosa 693(3), tongue 94, others 188	N/A	N/A	N/A	N/A	N/A	N/A	N/A	N/A	N/A	Yes	Described	No	Clinic (and histopathology in 13% of cases)	No	No	F:364(0) M:358(3)	N/A (32-38-47)	+91 (+3) -631 (-0)	N/A	N/A	N/A	N/A	0
Holmstrup *et al.*	1988	Denmark	Copenhagen University Hospital	P	1972-1985	12-312	Mean 90	N/A	611	9	1.47	121.2	N/A (tongue 5, gingiva 3, buccal mucosa 1)	N/A	N/A (2)	N/A (7)	N/A	N/A	N/A	N/A	N/A	N/A	Yes	Described	No	Clinic and histopathology	Yes	No	F:409(8) M:202(1)	N/A (56-63-65-70-70-76-76-79-79)	N/A (4)	N/A (+0)	N/A	N/A	N/A	0
Salem	1989	Saudi Arabia	King Fahd Central Hospital (Gizan)	R	1982-1987	≤60	Mean 38.4	Every 2 months	72	4	11.11	N/A	Buccal mucosa 55, tongue 31, lips 28, gingiva 12, palate 2 (N/A)	N/A	N/A	N/A	18	28	22	N/A	N/A	hypertrophic 4	No	Described	No	Clinic and histopathology	No	No	F:32 M:40 (N/A)	Range 18-72 years (N/A)	+32 (N/A)	N/A	N/A	+4 (N/A)	N/A	0
Vincent *et al.*	1990	USA	University of Iowa College of Dentistry	R	N/A	≤300	Mean 9.1	N/A	100	0	0	0	Buccal mucosa78, glossal mucosa44, gingiva42 (0)	N/A	N/A	N/A	N/A	N/A	N/A	N/A	N/A	N/A	Yes	Non described	No	Clinic	No	No	F:76(0) M:24(0)	Mean 64.2 years, range 18-90 (0)	N/A	N/A	N/A	N/A	N/A	0
Sigurgeirsson and Lindelöf	1991	Sweden	Karolinska Hospital (Stockholm)	R	1958-1987	≤348	Mean 118.8	N/A	2071	8	0.39	55.8	N/A (tongue 2, lip 3, floor of mouth 1,palate 1,unknown 1)	N/A	N/A	N/A	N/A	N/A	N/A	N/A	N/A	N/A	No	Non described	No	Clinic	No	No	F:1048(8) M:1023(8)	Mean 52 years, range 1-96 (29-35-61-67-69-76-77-83)	N/A	N/A	N/A	N/A	N/A	0
Silverman *et al.*	1991	USA	University of California School of Dentistry	P	N/A	6-420	Mean 90	N/A	214	5	2.34	108	Buccal mucosa 86(1), gingiva 68(2), tongue 46(2), palate 13, floor of mouth 12	N/A	N/A	N/A	61(1)	88 (3)	65 (1)	N/A	N/A	N/A	Yes	Described	No	Clinic or histopathology	Yes	No	F:152(4) M:62(1)	Mean 54 years, range 21-83 (N/A)	+12 (+1) -202 (-4)	N/A	N/A	+14 (N/A)	N/A	0
Voute *et al.*	1992	The Netherlands	Free University, Amsterdam	R	1970-1991	6-264	93.6	Bi-annual examination	113	3	2.65	84	N/A (buccal mucosa 2, tongue 1, lip 1)	N/A	N/A	N/A	N/A (1)	N/A (2)	N/A	N/A	N/A	N/A	Yes	Non described	No	Clinic or histopathology	No	No	F:79(2) M:34(1)	Mean 48.3 years, range 10-79 (48-58-65)	N/A (+8)	N/A	N/A	N/A	N/A	2 patients-single carcinoma 1 patient-2 carcinomas
Moncarz *et al.*	1993	Israel	Hadassah School of Dental Medicine (Jerusalem)	R	1984-1993	≤108	N/A	N/A	280	8	2.86	N/A	N/A (tongue 6, gingiva 3, buccal mucosa 2, floor of mouth 1, lip 1)	N/A	N/A	N/A	N/A	N/A	N/A	N/A	N/A	N/A	Yes	Described	No	Clinic or histopathology	No	No	N/A (F:6 M:2)	Mean 46 years, range 15-76 (mean 51 years, range 34-76)	N/A (+3)	N/A (+0)	N/A	N/A	N/A	6 patients-Sigle carcinoma 2 patients-multiple carcinomas
Barnard *et al.*	1993	UK	Bristol Dental Hospital & School	R	1982-1992	≤120	N/A	Bi-annual examination	241	9	3.73	69	N/A (tongue 6, buccal mucosa 1, gingiva 1, vestibulum 1)	N/A	N/A	N/A (2)	N/A	N/A (5)	N/A	N/A	N/A (2)	N/A	Yes	Described	No	Clinic and histopathology	No	No	N/A (F:4 M:5)	N/A (39-43-58-66-68-74-76-78-79)	N/A	N/A	N/A	N/A	N/A	0
Brown *et al.*	1993	USA	Georgetown University. Washington DC	R	1981-1987	24-108	N/A	N/A	193	0	0	0	Buccal mucosa>gingiva>tongue (0)	124	66	3	N/A	N/A	N/A	N/A	N/A	N/A	Yes	Non described	No	Clinic	No	No	F:138 M:55	Mean 54.9 years	N/A	N/A	N/A	N/A	N/A	0
Pang and Freeman	1995	Australia	Contact & Occupational Dermatitis Clinic of the Skin & Cancer Foundation (NSW)	R	1985-1994	≤108	N/A	N/A	19 LRs	1	5.26	N/A	Buccal mucosa 19, tongue 12, gingiva 3 (N/A)	N/A	N/A	N/A	14 (N/A)	7 (N/A)	N/A	N/A	6 (N/A)	N/A	No	Described	Yes (exclusion)	Clinic	No	No	F:17(1) M:2	Mean 52.6 years, range 28-72 (N/A)	N/A	N/A	N/A	N/A	N/A	1 patient-multiple carcinomas
Duffey *et al.*	1996	USA	UCLA Medical Center (Los Ángeles)	R	1987-1994	≤72	N/A	N/A	955	5	0.52	48.2	N/A (buccal mucosa 2, tongue 3, gingiva 1)	N/A	N/A	N/A	N/A	N/A (4)	N/A	N/A	N/A	N/A (unknown 1)	No	Non described	No	Clinic and histopathology	No	No	N/A (F:5)	N/A (62-62-68-70-70)	N/A	N/A	N/A	N/A	N/A	4 patients-single carcinoma 1 patient-2 carcinomas
Gorsky *et al.*	1996	Israel	School of Dental Medicine, Tel Aviv University	R	N/A	3-180	Mean 18	N/A	157	2	1.27	58.5	Buccal mucosa 123(2), gingiva 45, tongue 41, palate 10, floor of mouth 6, lips 4	N/A	N/A	N/A	66	54 (2)	26	N/A	N/A	unknown 11	Yes	Described	No	Clinic and histopathology	No	No	F:95(1) M:62(1)	Mean 53 years, range 22-89 (70-73)	+43 (+0) -114 (-2)	+3 (+0) -157 (-2)	N/A	+21 (N/A)	N/A	0
Markopoulous *et al.*	1997	Greece	Oral Medicine Clinic of the University of Thessaloniki	R	N/A	6-112	Mean 57.6	Anual or biannual examination	326	4	1.23	78	Buccal mucosa 258(1), tongue 103 (2), palate 34, lips 12(1), floor of mouth 24, gingiva 22	N/A	N/A	N/A	205	88 (3)	23	4	3	papular 3 (1)	Yes	Described	No	Clinic and histopathology	Yes	No	F:239(1) M:87(3)	Mean 54.8 years (26-50-60-74)	+60 (+0) -266 (-4)	N/A	N/A	+14 (N/A)	Nutritional disturbances were excluded	0
Silverman and Bahl	1997	USA	University of California School of Dentistry	R	1995-1996	12-240	N/A	N/A	95	7	7.37	95.28	N/A (primary sites: tongue 4, gingiva 2, lip 1, secondary site: oropharynx 1)	N/A	N/A	N/A	27	55 (3)	13 (4)	N/A	N/A	N/A	Yes	Non described	No	Clinic and histopathology	No	No	F:66(4) M:29(3)	F:mean 60 years, range 33-92 M:mean 56 years, range 33-79 (38-44-56-61-65-71-78)	+10 (+0) -85 (-7)	N/A	N/A	+7 (N/A)	N/A	6 patients-single carcinoma 1 patients-2 carcinomas
Girod *et al.*	1998	Germany	Department of Oral and Maxillofacial Surgery, University of Cologne	R	N/A	≥151	N/A	N/A	23	1	4.35	151	N/A (buccal mucosa 1)	N/A	N/A	N/A	N/A	N/A	N/A	N/A	N/A	N/A	Yes	Non described	No	Clinic and histopathology	No	No	N/A (M:1)	N/A	N/A	N/A	N/A	N/A	N/A	0
Lo Muzio *et al.*	1998	Italy	Division of Oral Medicine and Pathology, University of Naples 'Federico II'	R	1986-1996	12-120	N/A	Anual examination	263	14	5.32	64.32	N/A (buccal mucosa 12,tongue 7, gingiva 4, palate 4, tuber 2, vestibulum 5)	N/A (2)	N/A (14)	N/A	N/A	N/A	N/A	N/A	N/A	N/A	Yes	Described	No	Clinic and histopathology	No	No	F:156(5) M:107(9)	mean 55.5 years, range 22-80 (24-32-34-43-43-56-58-58-60-61-63-63-64-64)	+74 (+4) -189 (-10)	N/A (+0)	N/A (+3)	N/A (+2)	N/A	6 patients-single carcinoma 2 patients-2 carcinomas 3 patient-3 carcinomas 1 patient-4 carcinomas 1 patient-5 carcinomas 1 patient-6 carcinomas
Rajentheran *et al.*	1999	UK	Dental Hospital, Newcastle	R	1983-1996	≤312	N/A	N/A	832	7	0.84	72	N/A (buccal mucosa 4, gingiva 2, tongue 3, palate 1, floor of mouth 1)	N/A	N/A	N/A	N/A	N/A (5)	N/A (1)	N/A	N/A (1)	N/A	Yes	Described	No	Clinic and histopathology	Yes	No	F:524(5) M:308(2)	N/A (42-48-50-52-52-52-61)	N/A (+3)	N/A (+1)	N/A	N/A	N/A	5 patients-single carcinoma 1 patient-2 carcinomas 1 patient-4 carcinomas
Rode *et al.*	2000	Slovenia	Medical Center Ljubljana	R	1975-1994	≤228	N/A	Bi-annual examination	75	0	0	0	Buccal mucosa 47, retromolar region 15, tongue 10, floor of mouth and gingiva 3 (0)	N/A	N/A	N/A	75	N/A	N/A	N/A	N/A	N/A	Yes	Non described	No	Clinic and histopathology	No	No	F:60 M:15 (0)	Mean 58.3 years (0)	N/A	N/A	N/A	N/A	N/A	0
Chainani-Wu *et al.*	2001	USA	Oral medicine clinic, University of California	R	1996-2000	≤331	N/A	N/A	229	4	1.75	168	N/A (Lip 1, retromolar region 1, gingiva 1,maxillary tuberosity 1)	N/A	N/A	N/A	56 (N/A)	96 (N/A)	77 (N/A)	N/A	N/A	N/A	Yes	Non described	No	Clinic and/without histopathology	No	No	F:157 M:72 (N/A)	Mean 55 years (52-54-70-79)	+70 (N/A)	N/A	+31 (N/A)	+10 (N/A)	N/A	0
Mignogna *et al.*	2001	Italy	Division of Oral Medicine and Pathology, University of Naples 'Federico II'	R	1995-2000	4-144	N/A	Every 4 months	502	24	4.78	27.96	Buccal mucosa(10)>tongue(9)>gingiva(7)>palate(1)>lips>floor of mouth	N/A (2)	N/A (22)	N/A	N/A	N/A	N/A	N/A	N/A	N/A	Yes	Described	No	Clinic and histopathology	No	No	F:311(2 0) M:191(4)	Mean 55.4 years, range 18-83 (35-36-40-40-45-46-48-50-52-54-59-61-65-70-70-70-71-71-72-72-72-74-74-78)	N/A (+3)	N/A (+0)	N/A (+5)	N/A	Normal diet (fresh fruit and/or vegetables)	21 patients-single carcinoma 3 patient-2 carcinomas
Yaacob *et al.*	2002	Malaysia	Faculty of Dentistry, University of Malaya (Kuala Lumpur)	R	1980-2000	12-72	N/A	N/A	19	1	5.26	72	N/A (tongue 1)	5(1)	N/A	N/A	9	N/A	N/A	N/A	N/A	Unknown 5	Yes	Non described	No	Clinic (and non-systematic histopathological examination)	No	No	F:12(1) M:7	Mean 47.79 years, range 36-67 (47)	+3 (+0) -16 (-1)	+4 (+0) -15 (-1)	N/A	N/A	N/A	0
Bruno *et al.*	2002	Italy	Otorhinolaryngologic Department of the University of Rome «Tor Vergata»	R	1996-2000	12	N/A	Every 4 months	19	2	10.52	0	Buccal mucosa 2(1), tongue 5(1), vestibule 6, lips 2, palate 2, floor of mouth 1, retromolar trigone 1	N/A	N/A	N/A	5	6 (1)	3 (1)	1	2	papular 2	No	Non described	No	Clinic and histopathology	No	No	F:12 M:7 (N/A)	Mean 65.42 years, range 43-76 (N/A)	+19 (+2) -0 (-0)	+9 (N/A)	+1 (N/A)	+4 (N/A)	N/A	0
Bromwich	2002	UK	University Hospital of Wales, Cardiff, Wales	R	1996-2000	≤48	N/A	N/A	34	3	8.82	N/A	Buccal mucosa (1), tongue (2)	N/A	N/A (1)	N/A (1)	N/A	N/A (1)	N/A	N/A	N/A	N/A	No	WHO 1978	No	Clinic and histopathology	No	No	F: (2) M: (1)	N/A (66-75-76)	N/A	N/A	N/A	N/A	N/A	0
Eisen	2002	USA	Dermatology Research Associates, Mercy Health Plaza (Ohio)	R	N/A	6-96	Mean 54	N/A	723	6	0.83	40.9	Buccal mucosa 657 (1), tongue 435 (2), gingiva 401 (2), Lip 236, floor of mouth 77 (1), palate 43	N/A	N/A	N/A	267	290(4)	166 (2)	N/A	N/A	N/A	No	Non described	No	Clinic and histopathology	Yes	No	F:544(4) M:179(2)	F:mean 57 years M:mean 47 years (38-44-49-51-53-59)	N/A (+0)	N/A (0)	N/A	+36 (N/A)	N/A	0
Lanfranchi *et al.*	2002	Argentine	School of Dentistry University of Buenos Aires	R	1991-1997	2-72	N/A	N/A	491	32	6.52%	20.8	N/A (tongue 16, buccal mucosa 11, palate 3, alveolar ridge 2)	N/A	156 (8)	N/A (17)	N/A	210 (7)	61	37	N/A	pigmentary and pseudovegeta tive 27	Yes	Described	No	Clinic and histopathology	Yes	No	F:481(1 6) M:238(1 6)	mean 54,5 years, range 27-91 (29-30-41-44-45-46-47-49-50-50-56-56-59-60-60-60-60-61-66-67-67-68-69-71-71-73-74-75-77-78-81-83)	N/A	N/A	N/A	N/A	N/A	0
Machado *et al.*	2004	Brazil	University of Sao	R	N/A	3-144	Mean	N/A	52	0	0	0	Buccal mucosa 44, tongue 27, gingiva 13,	N/A	N/A	N/A	45	20	23	N/A	7	N/A	Yes	Described	No	Clinic (and histopathology in 38% of cases)	No	No	F:33(0)	Mean 49.7	N/A	N/A	N/A	-7 (+0)	N/A	0

			Paulo				16.8						lip 9, palate 2<br>(0)																M:19(0)	years, range 17-75<br>(0)				-45 (-0)		
Rödstrom *et al.*	2004	Sweden	Faculty of Odontology, Göteborg University, Sweden	R	1978-1993	19.2-115.2	Mean 76.8	N/A	1028	5	0.49	77.7	N/A<br>(buccal mucosa 3, floor of mouth 1, vestibulum 1)	N/A	N/A	N/A	N/A	N/A	N/A	N/A	N/A	N/A	Yes	Described	No	Clinic (and histopathology in 62% of cases)	No	No	F:677(4)<br>M:351(1)	Mean 55 years<br>(63-64-68-82-89)	N/A	N/A	N/A	N/A	N/A	0
Mattila *et al.*	2004	Finland	Department of Oral Pathology and Radiology, University of Turku	R	1991-2002	7.2-117	Mean 63.5	N/A	70	5	7.14	32.8	Buccal mucosa 59, tongue 13, gingiva 7, lips 2<br>(Buccal mucosa 1, tongue 4)	N/A	N/A	N/A	N/A	14 (1)	67 (4)	N/A	N/A	N/A	Yes	Non described	No	Clinic and histopathology	No	No	F:53(4)<br>M:17(1)	F:mean 53.3<br>M:mean 52.7<br>(41-54-58-62-69)	N/A	N/A	N/A	N/A	N/A	0
Xue *et al.*	2005	China	Stomatological Hospital of Wuhan University	R	1963-2003	≤252	N/A	N/A	674	4	0.59	108	Buccal mucosa 503(3)<br>Tongue 418(1)<br>Lips 311<br>Gingiva 205<br>Palate 17<br>Floor of mouth 13	N/A	N/A	N/A	346	263 (3)	65 (1)	N/A	N/A	N/A	Yes	Van der Meij 2003	Yes<br>(LR exclusión)	Clinic and histopathology	Yes	No	F:444(1)<br>M:230(3)	Mean 50.4 years, range 10-78<br>(35-50-57-65)	+174 (+3)<br>-500 (-1)	+162(+3)<br>-512(-1)	N/A	+78 (+0)<br>-596 (-4)	N/A	0
Laeijendecker *et al.*	2005	The Netherlands	Department of Dermatology, Erasmus MC, University Medical Center, Rotterdam and Department of Dermatology, Albert Schweitzer Hospital, Dordrecht	R	1991-2003	84-156	Mean 120	Anual or biannual examination	200	3	1.5	4.3	Buccal mucosa(1)>tongue(2)>gingiva>lips	N/A	92(1)	N/A	N/A	67 (2)	41	N/A	N/A	N/A	No	Described	No	Clinic and histopathology	Yes	No	F:132(2)<br>M:68(1)	mean 53 years, range 25–83<br>(59-67-78)	N/A<br>(+1)	N/A<br>(+0)	N/A	N/A	N/A	0
Roosaar *et al.*	2006	Sweden	Uppsala County	P	1973-2002	≤348	272.4	N/A	327<br>OLL	1<br>OLL	0.31	N/A	N/A	115<br>(N/A)	212<br>(N/A)	N/A	N/A	N/A	N/A	N/A	N/A	N/A	Yes	Described	No	Clinic	No	No	F:209(1)<br>M:118	15–24 20<br>25–34 38<br>35–44 48<br>45–54 77<br>55–64 83<br>65–74 41<br>75–84 17 (1)<br>>85 3	+135<br>-205<br>(N/A)	+22<br>-305<br>(N/A)	N/A	N/A	N/A	0
Bornstein *et al.*	2006	Switzerland	University Hospital of Berne	R	1995-2001	2.1-8.8	Mean 44.4	N/A	141	4	2.84	25.75	Buccal mucosa(4)> Gingiva>Tongue(2)	N/A	N/A	N/A<br>(4)	N/A	N/A	N/A	N/A	N/A	N/A	No	WHO 1978	Yes<br>(LR exclusion)	Clinic and histopathology	No	No	F:98(2)<br>M:47(2)	Mean 56.3 years<br>(52-60-69-69)	+18 (+1)<br>-127 (-3)	+52 (+0)<br>-93 (-4)	+1 (+1)<br>-144 (-3)	N/A<br>(+1)	N/A	3 patients-single carcinoma<br>1 patient-3 carcinomas
Ingafou *et al.*	2006	UK	Eastman Dental Institute, UCL.	R	N/A	3-240	N/A	N/A	690	13	1.88	N/A	Buccal mucosa>Tongue>Gingiva<br>(N/A)	N/A	N/A	N/A	651	16 (10)	256	27	225 (3)	Papular 78 (0)	Yes	Described	No	Clinic and histopathology	No	No	F:439(5)<br>M:251(8)	Mean 52 years, range 16–83<br>(median 65)	N/A	N/A	N/A	N/A	N/A	0
Hsue *et al.*	2007	Taiwan	Kaohsiung Medical University	R	1991-2001	6-120	N/A	N/A	143	3	2.10	14.67	N/A	N/A	N/A	N/A	N/A	N/A	N/A	N/A	N/A	N/A	Yes	Non described	No	Clinic and histopathology	No	Yes	N/A	N/A	N/A	N/A	N/A	N/A	N/A	0
van der Meij *et al.*	2007	The Netherlands	Department of Oral and Maxillofacial Surgery of the Vrije Universiteit Medical Center, Amsterdam	P	1996-2001	7.6-96.9	Mean 55.9 (whole group)<br>mean 55.9 (OLP group)<br>mean 53.8 (OLL group)	N/A	67 OLP<br>125 OLL	0 LPO<br>4 OLL	OLP:0<br>OLL:3.2	40	N/A<br>(Tongue 3<br>Gingiva 1)	N/A	N/A	N/A	N/A	N/A	N/A	N/A	N/A	N/A	Yes	Van der Meij 2003	Yes<br>(differentiation)	Clinic and histopathology	Yes	No	F:128(2)<br>M:64(2)	mean 52.5 years, range 17.5-79.2<br>(42-64-65-76)	N/A<br>(+1)	N/A<br>(+1)	N/A<br>(+0)	N/A<br>(+0)	N/A	0
Kesic *et al.*	2009	Serbia	School of Medicine, Nis	R	1997-2007	≤120	N/A	N/A	163	2	1.23	N/A	N/A<br>(Buccal mucosa 2)	N/A	N/A	N/A	80	42 (1)	40 (1)	1	N/A	N/A	Yes	Non described	No	Clinic and histopathology	No	No	F:114<br>M:49<br>(N/A)	Mean 53.90 years<br>(N/A)	N/A	N/A	N/A	+8<br>(N/A)	N/A	0
Fang *et al.*	2009	China	West China College of Stomatology, Chengdu.	R	1951-2006	≤660	N/A	N/A	2119	23	1.09	16	Buccal mucosa 1114<br>(Tongue 16<br>Buccal mucosa 4<br>Lips 2<br>Gingiva 1)	N/A<br>(22)	N/A<br>(11)	N/A	N/A	N/A	N/A	N/A	N/A	N/A	Yes	Van der Meij 2003	Yes<br>(LR exclusion)	Clinic and histopathology	Yes	No	F:1127(12)<br>M:992(11)	Mean 45 years<br>(30-37-40-41-42-42-44-44-47-48-52-53-55-55-56-56-60-63-63-65-65-65-68)	N/A<br>(+13)	N/A<br>(+12)	N/A	N/A	N/A	0
Pakfetrat *et al.*	2009	Iran	Oral Medicine Department of Mashhad Dental School	R	2000-2005	3-72	N/A	N/A	420	3	0.71	20	buccal mucosa(1)>tongue(2)>gingiva	146(3)	399(0)	N/A	N/A	N/A	N/A	N/A	N/A	N/A	Yes	Described	No	Clinic and histopathology	No	No	F:273(1)<br>M:147(2)	Mean 41.6 years, range 13-75<br>(N/A)	N/A<br>(+0)	N/A<br>(+0)	N/A	N/A	N/A	0
Ögmundsdóttir *et al.*	2009	Iceland	University of Iceland, Reykjavik	R	1985-1995	144–264	190.8	N/A	45	1	2.22	N/A	N/A<br>(tonsil 1)	N/A	N/A	N/A	N/A	N/A	N/A	N/A	N/A	N/A	No	Non described	No	Clinic and histopathology	No	No	F:17<br>M:28<br>(N/A)	Mean 52, range 23–81<br>(N/A)	N/A	N/A	N/A	N/A	N/A	0
Carbone *et al.*	2009	Italy	Turin Hospital	R	1987-2004	6-204	Median 47.4 (male group)<br>Median 44.8 (female group)	2-4-6-12 months-yearly	808	15	1.86	52.33	Buccal mucosa 590(8)<br>Tongue 355(6)<br>Gingiva 267(1)	332(9)	476(6)	N/A	N/A	N/A	N/A	N/A	N/A	N/A	Yes	Van der Meij 2003	Yes<br>(OLL and LR exclusion)	Clinic and histopathology	Yes	No	F:493(12)<br>M:315(3)	F:mean 61 years<br>M:mean 58 years<br>(49-52-54-64-65-67-67-67-69-71-72-73-75-80-80)	+179 (+5)<br>-629 (-7)	+99 (+3)<br>-709 (-9)	+137 (+4)<br>-671 (-8)	+65 (+1)<br>-743 (-11)	N/A	0
Oliveira *et al.*	2010	Brazil	São José dos Campos Dental School, São Paulo State University	R	1989-2009	≤240	N/A	N/A	110	0	0	0	Buccal mucosa 102<br>Tongue 50<br>Gingiva 21<br>(0)	N/A	N/A	N/A	90<br>(0)	46<br>(0)	N/A	N/A	N/A	N/A	No	Non described	No	Clinic and histopathology	Yes	No	F:84(0)<br>M:26(0)	Mean 53.8 years, range 22-97<br>(0)	+22 (+0)<br>+88 (-0)	+32 (+0)<br>-78 (-0)	N/A	+6 (+0)<br>-104 (-0)	N/A	0
Zyada *et al.*	2010	Egypt	Faculty of Dentistry, Mansoura University	R	2000-2007	24-84	N/A	N/A	43	3	6.98	N/A	Buccal mucosa 26<br>Tongue 10<br>Gingiva 5<br>Lip 1<br>Floor of mouth 1<br>(N/A)	15<br>(N/A)	19<br>(N/A)	9<br>(N/A)	N/A	N/A	N/A	N/A	N/A	N/A	Yes	Non described	No	Clinic and histopathology	No	No	F:28(2)<br>M:15(1)	56.7 years<br>(31-51-77)	N/A	N/A	+23(+3)<br>-20(-0)	N/A	N/A	0
Thongprasom *et al.*	2010	Thailand	Oral medicine clinics, Chulalongkorn and Mahidol (Central Thailand), Chiang Mai (Northern Thailand), and Khon Kaen (Northeastern Thailand) Universities	R	2002-2007	≥300	N/A	N/A	533	1	0.19	300	Buccal mucosa(1)>gingiva>tongue	N/A	N/A	N/A	N/A	N/A	N/A<br>(1)	N/A	N/A	N/A	Yes	Described	No	Clinic and/or histopathology	No	No	F:429(1)<br>M:104(0)	mean 50.9 years, range 17-90<br>(60)	N/A<br>(+0)	N/A<br>(+0)	N/A	+46<br>-587<br>(N/A)	N/A	0
Bajaj *et al.*	2010	India	Liaquat University Hospital, Hyderabad	R	2006-2007	≤24	N/A	N/A	95	0	0	0	Buccal mucosa 31 tongue 20<br>lips 28<br>palate 9<br>floor of mouth 3	N/A	N/A	N/A	52	31	12	N/A	N/A	N/A	No	Van der Meij 2003	Yes<br>(LR exclusion)	Clinic and/without histopathology	No	No	M:40(0)<br>F:55(0)	F:mean 34.41 years<br>M:mean 36.05 years<br>overall range 17-62 years.<br>(0)	+24 (+0)<br>-71 (-0)	N/A	N/A	+5 (+0)<br>-90 (-0)	N/A	0
Bermejo-Fenoll	2010	Spain	Department of Oral Medicine, University of Murcia. And private practice (Elche, Alicante)	R	1991-2007	≤192	N/A	N/A	550	5	0.91	N/A	Buccal mucosa 503<br>Gingiva 314<br>Tongue 209<br>Palate 74<br>Lips 81<br>Floor of mouth 33<br>(N/A)	359	191	N/A	N/A	N/A	N/A	N/A	N/A	N/A	Yes	WHO 1978	No	Clinic and histopathology	Yes	No	F:422(2)<br>M:128(3)	56.35 years, range 14–91<br>(N/A)	N/A<br>(+0)	N/A<br>(+0)	16<br>(0)	N/A	N/A	0
Torrente-Castells *et al.*	2010	Spain	Dental Clinic, University of Barcelona	R	1990-2005	10 days-212 months	Mean 18.2	N/A	65	2	3.08	N/A	buccal mucosa>tongue(2)>gingiva	22(2)	43	N/A	N/A	N/A	N/A	N/A	N/A	N/A	No	Van der Meij 2003	Not reported	Clinic and histopathology	Yes	No	F:40<br>M:25(2)	mean 59 years, range 16-88<br>(54-62)	+18 (+2)<br>-47 (-0)	+13 (+2)<br>-52 (-0)	N/A	N/A	N/A	0
Bombeccari *et al.*	2011	Italy	Department of Oral Pathology and Medicine, University of Milan	P	2001-2009	9-108	Mean 81.7	Every 4 months	327	8	2.45	39.38	N/A<br>(primary sites: tongue 5, buccal mucosa 3, secondary sites: buccal mucosa 2, alveolar ridge 3, tongue 2)	N/A<br>(3)	N/A<br>(4)	N/A<br>(1)	N/A	N/A	N/A	N/A	N/A	N/A	Yes	Van der Meij 2003	Yes<br>(OLL exclusion)	Clinic and histopathology	Yes	Yes	F:229(5)<br>M:98(3)	Mean 57.7<br>(55-63-67-68-68-68-69-71)	N/A<br>(+0)	N/A<br>(+0)	48+(0)<br>279-(8)	+28(+2)<br>-299 (-6)	N/A	5 patients-single carcinoma<br>2 patients- 3 carcinomas<br>1patient-4 carcinomas
Jaafari-Ashkavandi *et al.*	2011	Iran	School of Dentistry, Shiraz University of Medical Sciences	R	2006-2009	≤36	N/A	N/A	OLP:203<br>OLP with ED:14	OLP:2<br>OLP with ED:0	OLP:0.98<br>OLP with ED:0	N/A	buccal mucosa(1)>gingiva>tongue(1)	N/A	194(1)	N/A	N/A	17 (1)	N/A	6	N/A	N/A	Yes	Described	No	Clinic (and histopathology in 58.06% of cases)	No	No	217 casos,<br>H:M 1:2.3<br>(M:1,H:1)	Mean 46.7 years, range 11-84<br>(53-75)	N/A	N/A	N/A	N/A	N/A	0
Warnakulasuriya *et al.*	2011	UK	Guy's Hospital, London	R	1990-1999	6-180	N/A	N/A	OLP:607<br>LR:115<br>Mixed:175	OLP: 6<br>LR: 2<br>Mixed: 2	0.99	N/A	N/A	N/A	N/A	N/A	N/A	N/A	N/A	N/A	N/A	N/A	Yes	Described	No	Clinic and histopathology	No	No	N/A<br>(N/A)	N/A<br>(N/A)	N/A	N/A	N/A	N/A	N/A	0
Shen *et al.*	2012	China	Shanghai Jiao Tong, University School of Medicine	R	1978-2009	6-258	Mean	N/A	518	5	0.96	70.4	Buccal mucosa 455(2), tongue 295(1), lip 77(1), gingiva 26(1), palate 13, floor of mouth 3	247(4)	271(1)	N/A	N/A	N/A	N/A	N/A	N/A	N/A	Yes	WHO 1978	No	Clinic and histopathology	No	No	F:353(5)<br>M:165(0)	46.3 years, range 9-81<br>(46-48-50-51-58)	+43 (+0)<br>-475 (-5)	+41 (+0)<br>-477 (-5)	N/A	+7<br>-511<br>(N/A)	N/A	0
Kaplan *et al.*	2012	Israel	Department of Oral and Maxillofacial Surgery, Rabin Medical Center, Petah-Tiqva	R	1996-2007	12-192	Mean 51.6	every 4-6 months	171	10	5.84	N/A	Buccal mucosa(2)>tongue(4)>gingiva(3)>floor of mouth(1)	N/A	58(3)	36(7)	N/A	3	1	N/A	N/A	N/A	Yes	Van der Meij 2003	Yes<br>(OLL/LR exclusion)	Clinic and histopathology	No	No	M:51<br>F:120<br>(N/A)	mean 59.1 years, range 28–90<br>(N/A)	N/A	N/A	N/A	+23<br>(N/A)	N/A	0
Brzak *et al.*	2012	Croatia	Department of Oral Medicine, School of Dental Medicine, University of Zagreb	R	1998-2007	≤112	N/A	N/A	538	2	0.37	N/A	N/A (buccal mucosa and vestibular mucosa)	N/A	N/A	N/A	351	67	72	0	30	Papular 17<br>Unknown 1	Yes	Non described	No	Clinic and/or histopathology	No	No	F:379(1)<br>M:159(1)	N/A<br>(N/A-60)	+96<br>-413<br>29<br>unknown	N/A	N/A	N/A	N/A	0
Tovaru *et al.*	2013	Romania	Faculty of Dental Medicine, Carol Davila University, Bucharest	R	1990-2010	≤112	N/A	N/A	633	6	N/A	N/A	buccal mucosa> tongue>gingiva>lips> floor of mouth.<br>(N/A)	313(5)	310(1)	N/A	N/A	N/A	N/A	10 (0)	N/A	N/A	Yes	Van der Meij 2003	Yes<br>(LR exclusion)	Clinic and histopathology	No	No	F:498(4)<br>M:135(2)	F:mean 52 years<br>M:mean 64 years<br>(63-67-69-70-70-77)	N/A	N/A	61+(1)<br>572-(5)	+63 (+1)<br>-570(-5)	N/A	0
Gumru	2013	Turkey	University Faculty of Dentistry, Istanbul	R	1990-2010	≤112	N/A	N/A	330	1	0.30	24	Buccal mucosa 326(1)<br>Gingiva 96<br>Alveolar ridge 20<br>Tongue 102<br>Lips 30<br>Palate 34<br>Floor of mouth 11	224(1)	146	N/A	N/A	N/A	N/A	N/A	N/A	N/A	Yes	Van der Meij 2003	Yes<br>(LR exclusion)	Clinic and histopathology	Yes	No	F:260(1)<br>M:110	49.84 years, range 16-83<br>("an elderly woman")	+60 (+0)<br>-270 (-1)	+4 (+0)<br>-326 (-1)	N/A	+36<br>(N/A)	N/A	0
Bardellini *et al.*	2013	Italy	Oral Medicine Department of the Dental Clinic of the University of Brescia	R	1999-2010	6-144	N/A	½ month, 1 month, 6 months, 12 months, yearly	204	2	0.98	50	Buccal mucosa 177(1)<br>Tongue 171<br>Gingiva 86(1)<br>Palate 81	N/A	N/A	N/A	95	21 (2)	33	2	47	Papular 6	Yes	Van der Meij 2003	Yes<br>(OLL/LR exclusion)	Clinic and histopathology	Yes	No	M:41<br>F:163(2)	Mean 59, range 21-86<br>(57-63)	+14 (+0)<br>-190 (-2)	+10<br>-194<br>(N/A)	+46(+2)<br>-158 (-0)	+5(+0)<br>-199 (-2)	N/A	0
Wang *et al.*	2014	Taiwan	Kaohsiung Medical University Hospital	R	2001-2010	≤112	N/A	Every 3-6 months	381	2	0.52	8.07	Buccal mucosa 279<br>Gingiva 53<br>Tongue 22<br>Lower lip 20<br>(N/A)	N/A	N/A	N/A	N/A	N/A	N/A	N/A	N/A	N/A	Yes	Non described	No	Clinic and histopathology	No	No	M:149<br>F:232<br>(N/A)	Mean 51.08<br>(N/A)	+334<br>-36<br>(N/A)	+272<br>-108<br>(N/A)	N/A	N/A	N/A	0

Budimir *et al.*	2014	Croatia	Department of Oral Medicine, School of Dental Medicine, University of Zagreb	R	2006-2011	≤112	N/A	N/A	563	4	0.71	91.2	Buccal mucosa 469<br>Gingiva 111<br>Tongue 92<br>(N/A)	N/A	N/A	N/A	365	129 (2)	24 (1)	N/A	32 (1)	papular 13	Yes	Described	No	Clinic (and histopathology in 38% of patients)	No	No	M:149 (0) F:414(4)	67.12 years (mean 68.5 years)	+119 (+1) -433 (-3)	+27 -523 (N/A)	N/A	+29 (N/A)	N/A	0
Mostafa and Ahmed	2015	Egypt	Faculty of Oral and Dental Medicine, Cairo University	R	2012-2014	≤24	N/A	Every 3 months	64	2	3.12	N/A	Lips 28<br>Gingiva 37<br>Buccal mucosa 52<br>Tongue 11<br>Palate 2<br>(N/A)	N/A	N/A	N/A	6	13 (1)	38 (1)	3	2	papular 2	Yes	Van der Meij 2003	Yes (LR exclusion)	Clinic and histopathology	No	No	M:20 F:44 (N/A)	20-29:4<br>30-39:8<br>40-49:17<br>50-59:18<br>60-69:14<br>≥70:3<br>(N/A)	+20 (N/A)	N/A	11 (N/A)	+10 (N/A)	N/A	0
Casparis *et al.*	2015	Switzerland	Clinic of Oral Surgery, University of Zurich	R	2002-2011	≤112	N/A	N/A	OLP: 381 OLP OLL: 102 OLP with ED: 22	OLP 5 OLL 5 OLP with ED 2	OLP: 1.31 OLL: 4.90 OLP with ED : 9.09	18.96	Buccal mucosa 275 Tongue 81 Ridge 115 Floor of mouth 17 Palate 17 (OLP:palate 1, buccal mucosa 1, ridge 2, tongue 1 /OLL: ridge 2, buccal mucosa 2, tongue 1/ Dysplastic LP: ridge 1, tongue 1)	33	155(5)	N/A	69	122 (5)	N/A	N/A	N/A	lichenoid 126 (2)	Yes	Non described	Differentiation	Clinic and histopathology	No (different groups)	No	F:310 M:195 (OLP: M:2 F:3/OLL: 2 M:3/ Dysplastic LP: F:2 M:0)	Median 58 years, Range 15–98 (OLP:36-43-51-71-74/OLL:47-50-65-73-83/ Dysplastic LP:77-86)	N/A	N/A	N/A	N/A	N/A	0
Irani *et al.*	2016	Iran	Department of Pathology, The Educational Hospital, Hamadan	R	2005-2012	≤84	N/A	N/A	112	1	0.89	3	buccal mucosa 52<br>gingiva 26<br>labial mucosa 15 tongue 15 (1)<br>floor of mouth 4	N/A	N/A	N/A	28	50	8	N/A	23	papular 1	Yes	Van der Meij 2003	Yes (OLL exclusion)	Clinic and histopathology	No	Yes	M:39 F:73 (1)	15-86 years, mean 44.5 (61)	N/A (+0)	0	N/A	N/A	N/A	0
Varghese *et al.*	2016	India	Mar Baselios Dental College and Hospital, Kerala	R	2014-2015	≤24	N/A	N/A	122	0	0	0	Bilateral buccal mucosa 59<br>Right buccal mucosa 10<br>Left buccal mucosa 2<br>Palate 2<br>Tongue 6	N/A	N/A	N/A	75	38	N/A	6	4	N/A	Yes	Van der Meij 2003	Yes (OLL exclusion)	Clinic and histopathology	No	No	F:79(0) M:43(0)	15-30:8(0) 30-60:85(0) >60:29(0)	+6 (+0) -116 (-0)	+6 (+0) -116 (-0)	N/A	+11(+0) -111(-0)	N/A	0
Lauritano *et al.*	2016	Italy	Dental Clinic, University of Bari	R	2011-2014	≤36	N/A	N/A	87	1	1.49	60	Buccal 56 (1 left cheek)<br>Tongue 31<br>Gums 20<br>Hard palate 7<br>Fornix 6<br>Lip 6<br>Soft palate 3	34(N/A)	53(N/A)	N/A	N/A	N/A	N/A	N/A	N/A	N/A	No	Van der Meij 2003	Not reported	Clinic and histopathology	Yes	No	F:56(0) M:31(1)	59.15 years (N/A)	N/A	N/A	N/A	+21 (N/A)	N/A	0
Yahalom *et al.*	2016	Israel	Oral Medicine Clinic, Department of Oral and Maxillofacial Surgery, The Chaim Sheba Medical Center, and Tel-Hashomer	P	N/A	12-82	43.46	Biannual examination	30	3	10	32.44	(gingiva lower 2, buccal mucosa 1)	N/A	N/A	N/A	12 (2)	7	11(1)	N/A	N/A	N/A	Yes	WHO 1978	No	Clinic and histopathology	No	No	F:20(1) M:10(2)	mean age 56.6 years (60-62-65)	+6 (+0) -24 (-3)	N/A	N/A	N/A	N/A	0
Rimkevičius *et al.*	2017	Lithuania	Vilnius University Dental Clinic	R	2009–2013	≤48	N/A	N/A	133	3	2.25	N/A	Buccal mucosa 114<br>Gingival 61<br>Tongue 42<br>Lips 12<br>Vestibular 11<br>Palate 5<br>(N/A)	N/A	N/A	N/A	58	47 (3)	15	2	N/A	papular 11	No	Described	No	Clinic and histopathology	No	No	F:111(N/A) M:25(N/A)	N/A	+14 (N/A)	+3 (N/A)	N/A	N/A	N/A	0
Bandyopadhyay *et al.*	2017	India	Department of Oral Pathology and Microbiology, Kalinga Institute of Dental Sciences, Bhubaneswar, Odisha	R	2010-2016	≤72	N/A	N/A	143	2	1.40	42	Buccal mucosa (bilateral) 114 (2)<br>Buccal mucosa tongue 3<br>Left alveolar mucosa 4<br>Left buccal mucosa 4<br>Lower labial mucosa 4<br>Right buccal mucosa 9<br>Tongue 5	N/A	N/A	N/A	113	30 (2)	N/A	N/A	N/A	N/A	Yes	WHO 1978	No	Clinic and histopathology	No	Yes	F:65(1) M:78(1)	18-27: 32<br>28-37: 44<br>38-47: 33<br>48-57: 17<br>58+: 17<br>(55-69)	N/A	N/A	N/A	+19 (+0)	N/A	0
González-Moles *et al.*	2017	Spain	Private Oral Medicine Clinic, Granada	R	2006-2016	≤120	N/A	2 months-6 months-12 months-yearly	21 OLP 81 OLL	3 OLP 1 OLL	OLP:14.28 OLL:1.23	9.72	Upper gingiva 32<br>Lower gingiva 30(2)<br>Left buccal mucosa 61<br>Right buccal mucosa 79(1)<br>Left vestibular sulcus 24<br>Right vestibular sulcus 9<br>Tongue 25(1)<br>Soft, hard palate, and pillars 7<br>Upper lip 7<br>Lower lip 9<br>Floor of mouth 0(2)	N/A	N/A	N/A	102	50 (4)	72	N/A	2	N/A	Yes	Van der Meij 2003	Yes (differentiation)	Clinic and histopathology	Yes	No	F:76(2) M:26(2)	mean 58 years, range:32–84 (39-50-54-72)	N/A	N/A	N/A	N/A	N/A	3 patients-single carcinoma<br>1 patient-3 carcinomas
Laniosz *et al.*	2018	USA	Olmsted County population, Minnesota	R	1986-2010	0.24-68.4	Median 34.8	N/A	303	7	2.31	Median 34.8	buccal mucosa (2) tongue (4) gingiva (1) hard palate (1)	N/A	N/A	9	170 (1)	79 (5)	1	6	38 (1)	N/A	No	Van der Meij 2003	Yes (OLL/LR exclusion)	Clinic and/or histopathology	No	No	F=197 M=106 (N/A)	Mean 55.4 (65.8 years, range, 33.1–76.0 years)	Never 157 Former 108 Current 38 (N/A)	None 102 Occasional 159 Frequent 42 (N/A)	+5 (N/A)	N/A	N/A	7 patients-single carcinoma<br>1 patient-2 carcinomas
Rock *et al.*	2018	Canada	BC Oral Biopsy Service, Vancouver General Hospital	P	1997-2013	13.2-168.3	Median 63	N/A	OLP with ED 73	6	8.22	Median 38.3	N/A	N/A	N/A	N/A	N/A	N/A	N/A	N/A	N/A	N/A	Yes	Described	Yes (exclusion)	Clinic and histopathology	Yes	No	F:43 M:30 (N/A)	Mean 56 years (N/A)	+45 (N/A)	N/A	N/A	N/A	N/A	0
Park *et al.*	2018	South Korea	Section of Dentistry of Seoul National University Bundang Hospital	R	2003-2017	≤168	Mean 26.88	N/A	113	0	0	0	mucosal (buccal mucosa, vestibule, and tongue) 78(0)<br>Gingival 10(0)<br>Mixed 25(0)	N/A	N/A	97	N/A	8	N/A	N/A	N/A	N/A	Yes	Described	No	Clinic (4 non-systematic biopsies)	No	No	F=79(0) M=34(0)	Range 31-91, mean 49.52 (0)	N/A	N/A	N/A	N/A	N/A	0
Kaomongkolgit et al.	2019	Thailand	Oral Medicine Clinic, Dental Hospital, Naresuan University	R	2002-2018	≤192	N/A	N/A	102	0	0	0	Buccal mucosa>gingiva>lips>tongue>tongue >palate>floor of mouth	N/A	N/A	N/A	89	45	95	0	6	papular 6	Yes	Van der Meij 2003	No	Clinic (48 non-systematic biopsies)	No	No	F=75(0) M=27(0)	Range 20-81 mean 56.4 (0)	+2 (0) -100 (0)	+14 (0) -88 (0)	N/A	+8(0) -94(0)	N/A	0
Gomez-Armayones et al.	2019	Spain	Bellvitge University Hospital and Odontological University Hospital of Barcelona	R	1995-2014	≤228	N/A	N/A	314	25	N/A	N/A	N/A	N/A	N/A	N/A	N/A	N/A	N/A	N/A	N/A	N/A	No	Non described	No	Clinic	Yes	No	N/A	N/A	N/A	N/A	N/A	N/A	N/A	N/A
Shearston et al.	2019	Australia	private oral medicine clinic	R	2006-2014	18-96	N/A	N/A	OLP 206 OLL 31 OLP with ED 44	OLP 1 OLL 0 OLP with ED 3	0.49 OLP 0 OLL 6.82 OLP with ED	57.6 OLP 0 OLL 55.2 OLP with ED	OLP: Buccal mucosa (1)>gingiva<br>OLL:Buccal mucosa>tongue>gingiva<br>Dysplatic LP:Buccal mucosa>tongue (2)>gingiva (1)	N/A	N/A	N/A	N/A	N/A	N/A	N/A	N/A	N/A	Yes	WHO blue book	Yes (differentiation)	Clinic and histopathology	Yes	No	OLP: F=147 (1) M=59(0) Dysplastic LP: F=27(3) M=17(0)	OLP (76) Dysplastic LP (59-62-72)	OLP: +46 (+0) -160 (-1) Dysplastic LP: +12(+0) -32(-3)	OLP: +18 (+0) -188 (-1) Dysplastic LP: +4(+0) -40(-3)	N/A	OLP: +16 (+1) -190 (-0) Dysplastic LP: +1+0) -43(-3)	N/A	N/A
Cozzani et al.	2019	Italy	Department of Dermatology and at the Department of Otorhinolaryngology-H&NSurgery of San Martino Polyclinic Hospital, University of Genoa	R	2015-2018	≤36	N/A	1-3-6 months and biannually	32	2	6.25	N/A	N/A	14 (1)	17 (1)	0	10 (1)	13 (1)	1	N/A	6	Papular 1	No	Non described	No	Clinic and histopathology	No	No	F=20(1) M=11(1)	Range 49-81 mean 56.4 (50-80)	+46 (+1) -160 (-1)	+46 (+1) -160 (-1)	(0)	(N/A)	N/A	N/A
Aguirre-Urizar et al.	2020	Spain	Oral Medicine and Oral and Maxillofacial Department of Stomatology II. University of the Basque Country (UPV/EHU).	A	N/A	36-229	Mean 71.73	N/A	299 OLP 85 OLL	5 OLP 5 OLL	1.70 OLP 5.90 OLL	51.8 OLP 50.8 OLL	Buccal mucosa 351 (3)<br>Tongue 241 (6)<br>Gingiva 193 (4)<br>Other 84 (1)	493	263	N/A	384	101	162	0	109	0	Yes	Described	Yes (differentiation)	Clinic and histopathology	No	No	F=272 (8) M=112 (2)	Range 21-90 Mean 57.15 years (54-56-57-60-65-67-69-72-74-76)	+78 (+2) -306 (-8)	+158 -226	N/A	+29 -355	N/A	8 patients-single carcinoma<br>2 patients-3 carcinomas
Guan et al.	2020	New Zealand	Oral Pathology Centre, University of Otago (Dunedin)	R	2005-2018	≤168	N/A	N/A	829 OLP/OLL	23	2.77	51.60	Buccal mucosa>gingiva>tongue	344	485	N/A	N/A	N/A	N/A	N/A	N/A	N/A	Yes	WHO 2003	No (OLR exclusion)	Clinic and histopathology	No	No	F=548 (21) M=281 (2)	Range 55-82 Mean 57.30 years (55,56,57,58, 58,60,60,62,62,62,64,64,68, 69,70,72,75,79,79,80,81,81, 82)	+116 (0) -713 (23)	+196 (0) -633 (23)	+2 -827 (N/A)	N/A	N/A	N/A
Dave et al.	2020	USA	Range 21-90 Mean 57.15 years	R	2000-2013	60-156	N/A	N/A	156	6	3.85	M/A	Buccal mucosa>gingiva>tongue	N/A	N/A	N/A	N/A	N/A	N/A	N/A	N/A	N/A	Yes	WHO 2003	Yes (OLL exclusion)	Clinic and histopathology	Yes	No	F=118 (4) M=38 (2)	Range 20-89 Mean 61 Years (range 50-80)	+13 -143 (N/A)	+66 -90 (N/A)	+3 -153 (N/A)	+35 -121 (N/A)	N/A	N/A
Tsushima et al.	2021	Japan	faculty of dentistry of Tokyo Medical and Dental University	R	2001-2017	6-220	Mean 55.9	N/A	565	4	0.7	52.5	Gingiva 310 (2)<br>Buccal mucosa 499 (1)<br>Tongue 96 (1)<br>Lip 43<br>Palate 23	325 (3)	240 (1)	0	N/A	N/A	N/A	N/A	N/A	N/A	No	AAOMP	Yes (differentiation)	Clinic and histopathology	Yes	No	F=442 (3) M=123 (1)	Mean 60.5 Range 21-93 (N/A)	+ N/A (0) - N/A (4)	+ N/A (0) - N/A (4)	+ 25 (0) - 540 (4)	+ 40 (0) - 525 (4)	N/A	0
Radochová et al	2021	Czech Republic	Oral Medicine Unit, Department of Dentistry Clinic Charles, University, Prague. Faculty of Medicine and University Hospital, Hradec Králové	R	2003-2020	≤216	Mean 15.2	N/A	271	2	0.74	65.45	Buccal mucosa 248 (1)<br>Gingiva 97<br>Tongue 152 (1)<br>Palate 20<br>Floor of mouth 12	295 (2)	284 (0)	0	259	173	120	2	111	0	No	Described	Yes (differentiation)	N/A	No	No	F=180 (1) M=91 (1)	Mean 56.0 Range 18.2-85.0	+52 (1) -219 (1)	N/A	N/A	+38 - 233 (N/A)	N/A	0
Arduino et al.	2021	IItaly	Department of Surgical Science, Oral Medicine Section, CIR-Dental School, University of Turin	R	1988-2020	12-384	48	N/A	3173	82	2.58	103.61	Buccal mucosa 2748 (70)<br>Tongue 1825 (47)<br>Gingiva 1296 (23)<br>Floor of mouth 103 (3)<br>Palate 381 (7)<br>Lips 270 (3)	1280 (41)	1893 (41)	0	N/A	N/A	N/A	N/A	N/A	N/A	Yes	Van der Meij 2003	Yes (OLL excluded)	Clinic and histopathology	Yes	No	M=1172 (32) F=2001 (50)	Mean 58.95 (60.78)	+441 (7) -2738 (75)	N/A	+193 (10) -2980 (72)	N/A	N/A	N/A
Migliari et al.	2022	Brazil	Dental School of the University of São Paulo	R	1997-2011	4	Mean 201	N/A	370	1	0.27	8	N/A (tongue)	N/A	N/A	N/A	N/A	N/A	N/A	N/A	N/A	N/A	No	Van der Meij 2003	Yes (OLL excluded)	Clinic and histopathology (partial, 58.1%)	Yes	No	M=93 F=277 (1)	Mean 54.8 Range 25-95	+66 -264 (N/A)	+63 -199 (N/A)	+7 -363 (N/A)	+68 -302 (N/A)	N/A	1
Xie et al.	2022	USA	Department of Dermatology, Mayo Clinic, Rochester, Minnesota	R	1995-2015	N/A	N/A	N/A	550	17	3.09	48	N/A (tongue 9, buccal mucosa 4, gingiva 4)	N/A	N/A	N/A	N/A (1)	N/A (10)	N/A	N/A	N/A (1)	N/A (5 unclear)	No	Van der Meij 2003 and AAOMP	Yes (OLL excluded)	Clinic and histopathology	Yes	No	N/A (M=7, F=10)	N/A (mean 65)	N/A (+4 -5)	N/A (+1 -8)	N/A (+0 -9)	N/A	N/A	N/A
Alkan et al.	2022	Israel	Department of Otorhinolaryngology, Rabin Medical Center, Petah-Tikva,	R	1995-2016	≤36		3-6 months, 2-4 visits	48	1	2.1	N/A	N/A (N/A)	N/A	N/A	N/A	N/A	N/A	N/A	N/A	N/A (1)	N/A	Yes	Described	Yes (OLL excluded)	Clinic and histopathology	No	No	M=17 F=31 (N/A)	Mean 55.5 Range 34-75 (N/A)	N/A	N/A	N/A	N/A	N/A	1
Cai et al.	2022	China	Department of Oral Pathology, Peking University Hospital of Stomatology	R	2010-2017	N/A	Mean 96	N/A	3568	10	0.28	54	Buccal mucosa 2254 (3)<br>Tongue 1135 (6)<br>Gingiva 112 (1)<br>Lip 66<br>Palate 1	N/A	N/A	N/A	N/A	N/A	N/A	N/A	N/A	N/A	Yes	Van der Meij 2003	Yes (OLL excluded)	Clinic and histopathology	Yes	No	M=1263 (4) F=2305 (6)	Mean 58 (61.5) Range 6-84	N/A (+1 -9)	N/A (+1 -9)	N/A (+0 -10)	N/A (+3 -)	N/A	21
Utz et al.	2022	Switzerland	Department of Dermatology at the University Hospital of Bern and the Department of Oral Surgery and Stomatology	R	2015-2020	2.1-66.5	19.6	4 visits first year, an then, 1 visits annually	57	4	7.02	N/A	Buccal mucosa 52<br>Tongue 22<br>Gingiva 42<br>Lips 9<br>Palate 7<br>(N/A)	50 (N/A)	7 (N/A)	N/A	N/A	N/A	N/A	N/A	N/A	N/A	Yes	Van der Meij 2003	Yes (OLL excluded)	Clinic and histopathology	Yes	No	M=15 (2) F=42 (2)	Mean 66 Range 33-99	N/A (+2 -2)	N/A (N/A)	N/A (N/A)	N/A (N/A)	N/A (N/A)	6
Kakoei et al.	2022	Iran	Oral Medicine Department of Dental School of Kerman Medical	R	1997-2020	N/A	N/A	N/A	294 OLP 62 OLL	9 OLP 0 OLL	3.06 OLP 0 OLL	N/A	N/A	N/A	N/A	N/A	N/A	N/A	N/A	N/A	N/A	N/A	No	Van der Meij 2003	Yes (OLL differentiataion)	Clinic and histopathology	No	No	M=97 (8) F=257 (14)	Mean 47	N/A (N/A)	N/A (N/A)	N/A	+17 - 337	N/A	N/A

			University																										Missing =2							
Saepoo et al.	2023	Thailand	Dental Hospital, Faculty of Dentistry, Prince of Songkla University	R	2016-2022	N/A	15.6	N/A	103 OLP OLR 14	2 OLP 0 OLR	1.94 OLP 0 OLR	180.5 OLP	N/A (N/A)	N/A	N/A	N/A	N/A	N/A	N/A	N/A	N/A	N/A	No	Van der Meij 2003 and Gonzalez-Moles and Ramos-Garcia 2022	Yes (OLL excluded)	Clinic and histopathology	No	No	M=92 F=25 overall	55.4 (overall)	N/A (+1 -1)	N/A (N/A)	N/A (+1 -1)	N/A (N/A)	N/A (N/A)	0
Obermeier et al.	2023	Germany	Departament of oral and maxillofacial surgery of Munich	R	2005-2022	N/A	Mean 80.52	N/A	103	17	16.50	3.1	N/A (N/A)	N/A	N/A	N/A	N/A	N/A	N/A	N/A	N/A	N/A	Yes	Non described	Yes (OLL excluded)	Clinic and histopathology	No	No	M=23 (2) F=80 (15)	N/A	N/A	N/A	N/A	N/A	N/A	17
Korkitpoonpol and Kanjanabuch	2023	Thailand	Oral Medicine Clinic, Faculty of Dentistry, Chulalongkorn	R	2012-2021	N/A	N/A	N/A	72 OLP 43 OLL 21 OLP with ED	1 OLP 0 OLL 0 OLP with ED	1.39 OLP 0 OLL 0 OLP with ED	84	N/A (Buccal mucosa 1)	N/A	N/A (1)	N/A	N/A	N/A	N/A	N/A	N/A (1)	N/A	Yes	Van der Meij 2003	Yes (OLL differentiataion)	Clinic and histopathology	Yes	No	OLP: M=13 (0) F=59 (1) OLL: M=4 (0) F=39 (0) OLP with ED: M=4 (0) F=17 (0)	OLP: 49.50 (64.0) OLL: 59.0 OLP dysp: 53.0	N/A	N/A	N/A	N/A	N/A	1
Tenore et al .	2023	Italy	Department of Oral Sciences and Maxillofacial Surgery, Sapienza University of Rome; Head and Neck Tumor Board, Umberto I University Hospital, Sapienza University of Rome	R	2015-2022	≤3	34.42 Range:4 -104	Mean 4 Range 1-8	100	11	1.10	N/A	Buccal mucosa 83 Tongue 62 Floor of mouth 17 Palate 43 Gingiva 59 (N/A)	28 (4)	72 (7)	0	N/A	6 (1)	22 (3)	N/A	N/A	N/A	Yes	AAOMP	Yes (OLL excluded)	Clinic and histopathology	Yes	no	M=41 (4) F=59 (7)	Mean 64.03 (74.91)	+:45 (6) -:55 (5)	+:15 (4) -:85 (7)	+:4 (1) -:96 (10)	+:12 (1) -:88 (10=	N/A	11

OLP, oral lichen planus; OLL, oral lichenoid lesion; LR, lichenoid reaction; ED, epithelial dysplasia; R, retrospective; P, prospective; A, ambispective; n, total cases; (M), cases with malignant transformation; +, positive cases; -, negative cases; HCV, hepatitis C virus; DM, diabetes mellitus; N/A, not available.

3. Meta-analyses on malignant transformation proportions

3.1 Subgroup meta-analysis stratified by OLP vs OLP with dysplasia

Figure S1. Forest plot graphically representing the meta-analysis of the malignant transformation proportion (expressed as percentage) stratified by studies that only include patients with oral lichen planus with epithelial dysplasia, and oral lichen planus (excluding or taking no account of the presence of epithelial dysplasia). Pooled proportions and 95% confidence intervals (CI) were used as effect size metric.

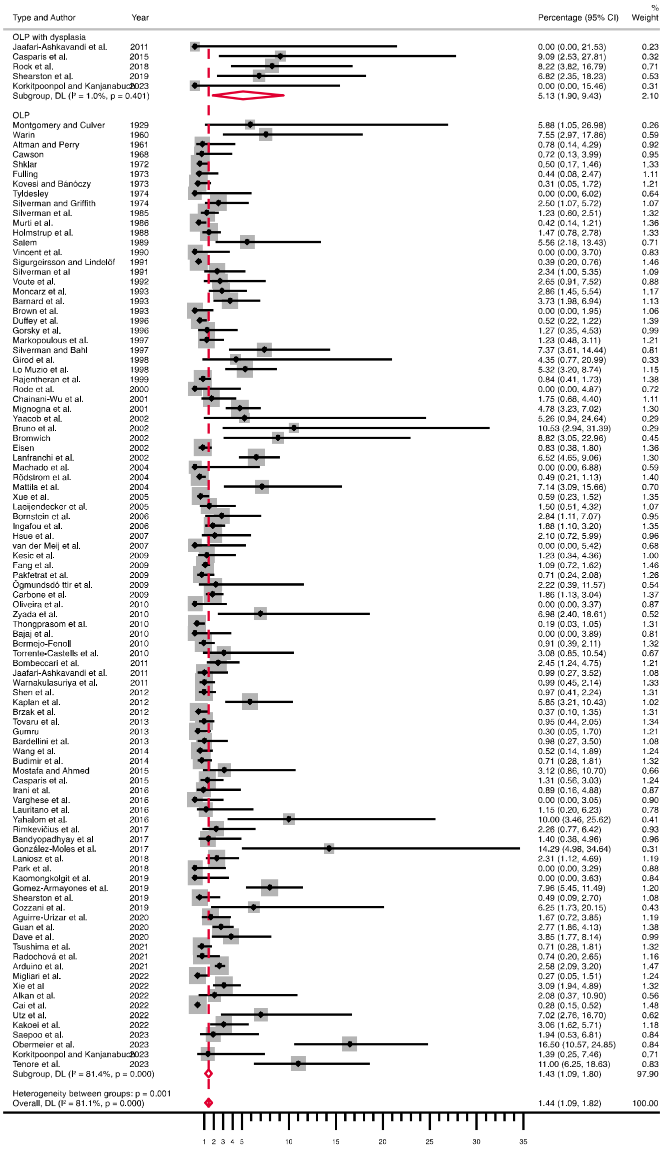

3.2 Subgroup meta-analysis stratified by oral lichenoid lesions and oral lichen planus.

Figure S2. Forest plot graphically representing the meta-analysis of the malignant transformation proportion (expressed as percentage) stratified by oral lichen planus and oral lichenoid lesions. Pooled proportions and 95% confidence intervals (CI) were used as effect size metric.

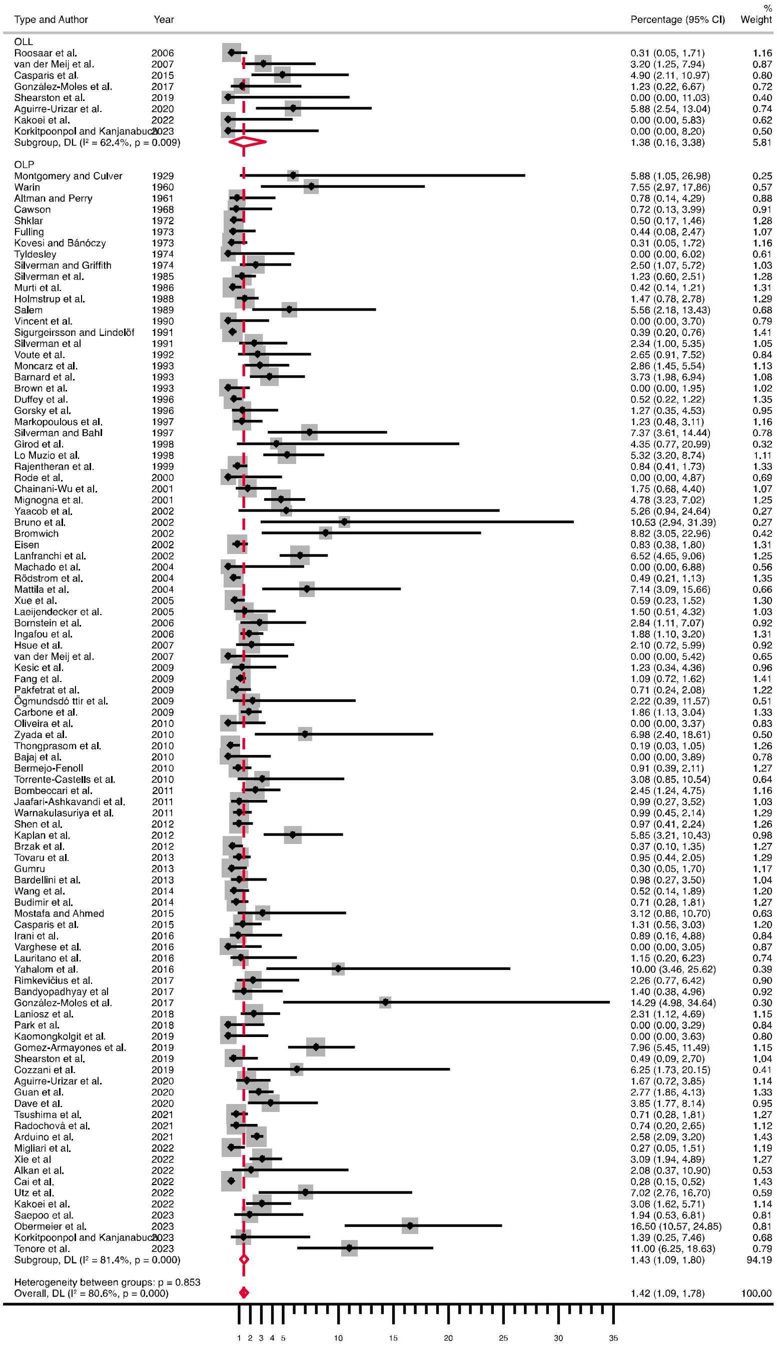

3.3 Subgroup meta-analysis stratified by lichenoid reactions and oral lichen planus.

Figure S3. Forest plot graphically representing the meta-analysis of the malignant transformation proportion (expressed as percentage) stratified by oral lichen planus and lichenoid reactions. Pooled proportions and 95% confidence intervals (CI) were used as effect size metric.

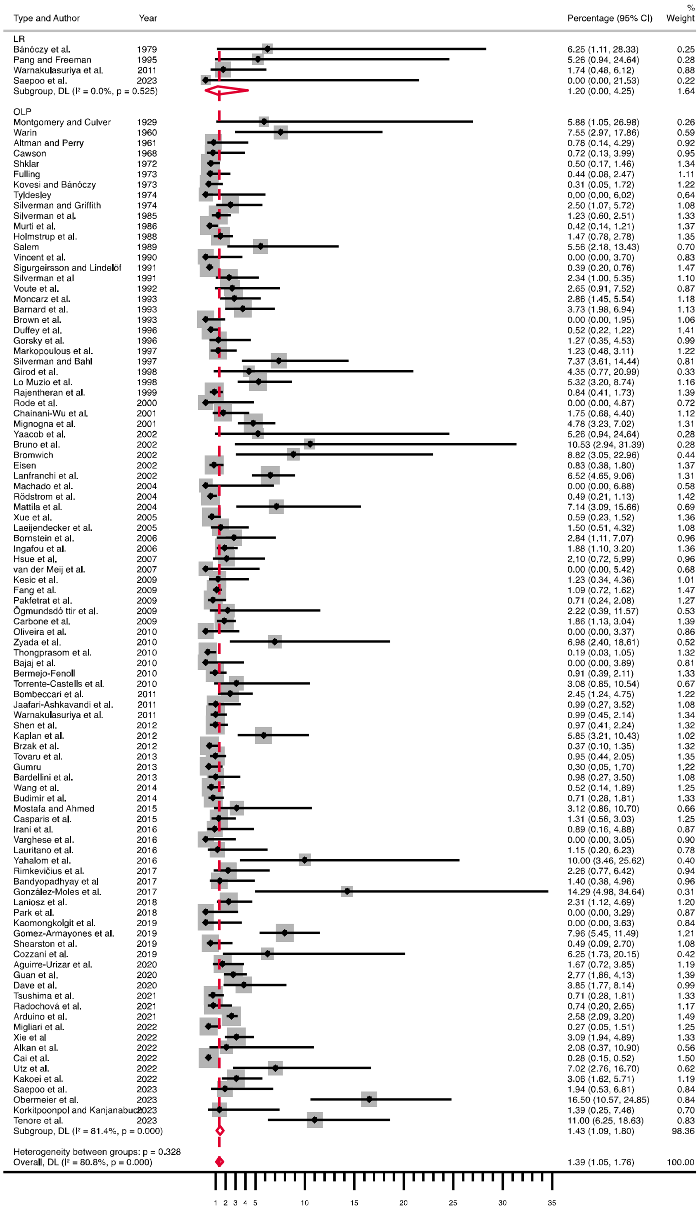

3.4 Subgroup meta-analysis stratified by Stratified by diagnostic criteria

Figure S4. Forest plot graphically representing the meta-analysis of the malignant transformation proportion (expressed as percentage) stratified by the presence or absence of exhaustive clinical and histopathological diagnostic criteria. Pooled proportions and 95% confidence intervals (CI) were used as effect size metric.

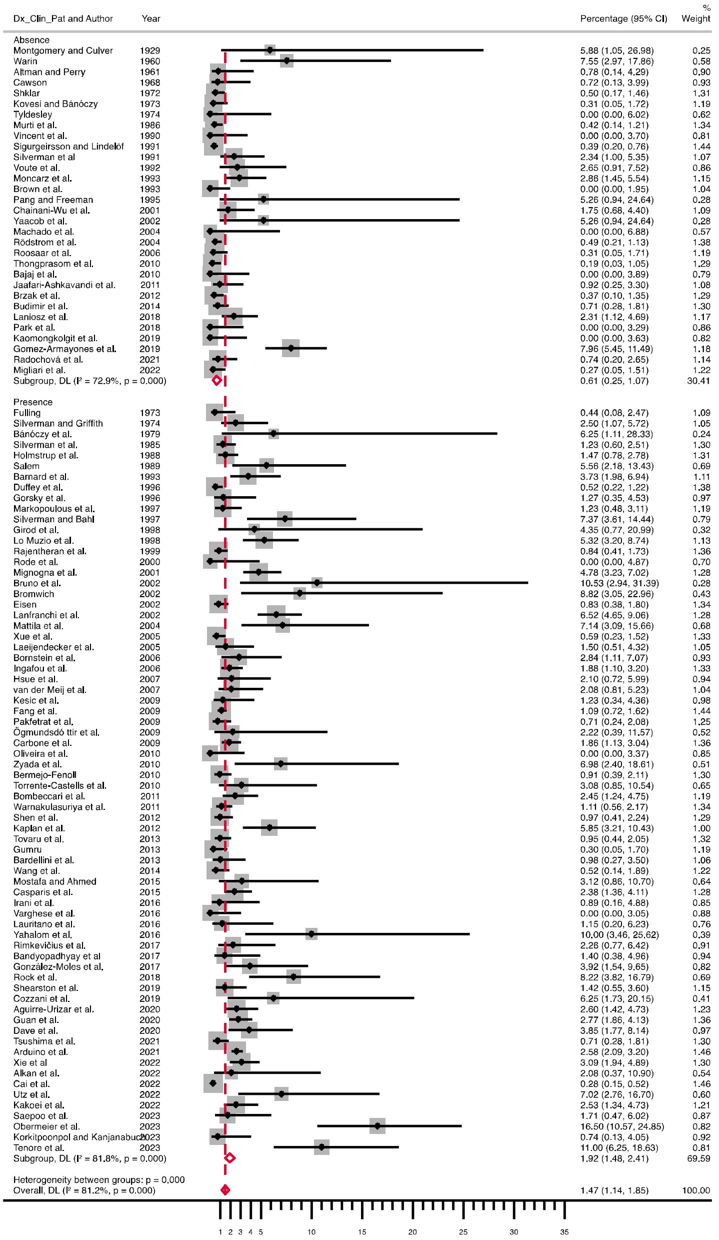

4. Sex and malignant transformation

Figure S5. Forest plot graphically representing the prognostic meta-analysis of the risk of oral cancer development in males *versus* females with OLP. Relative risk (RR) and 95% confidence intervals (CI) were used as effect size metric.

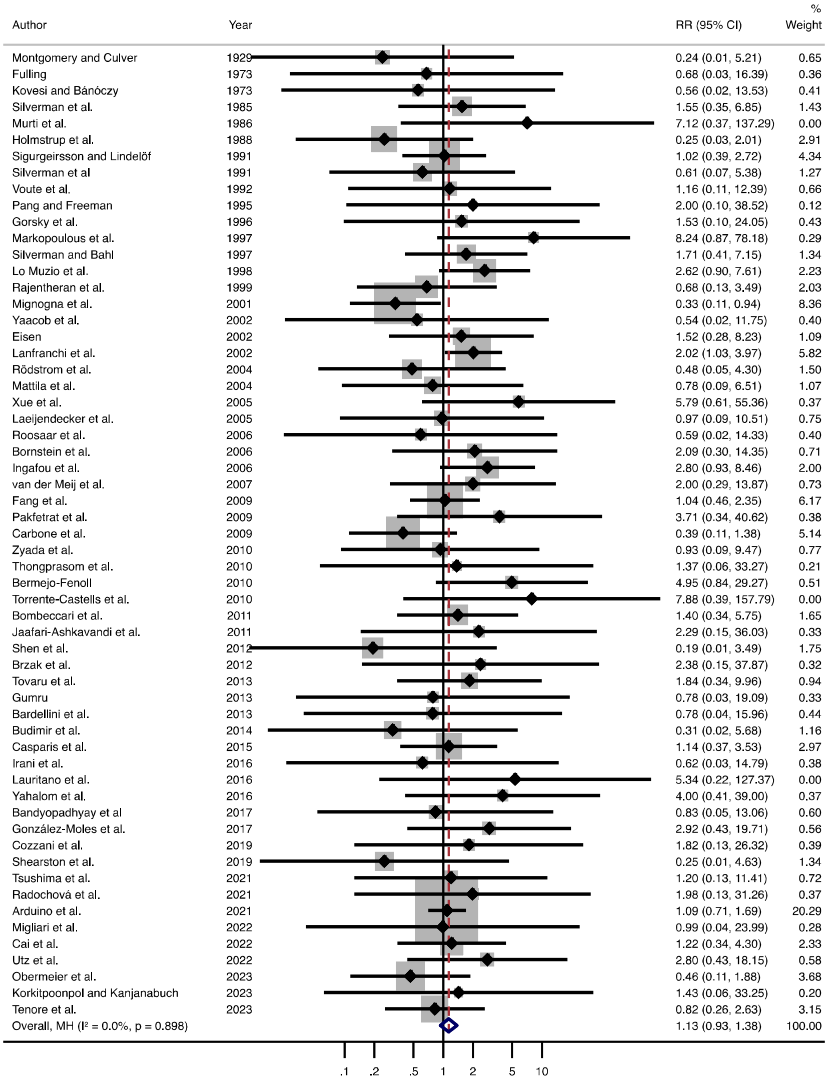

5. Clinical appearance of lesions and malignant transformation

Figure S6. Forest plot graphically representing the prognostic meta-analysis of the risk of oral cancer development in red OLP (vs. white). Relative risk (RR) and 95% confidence intervals (CI) were used as effect size metric.

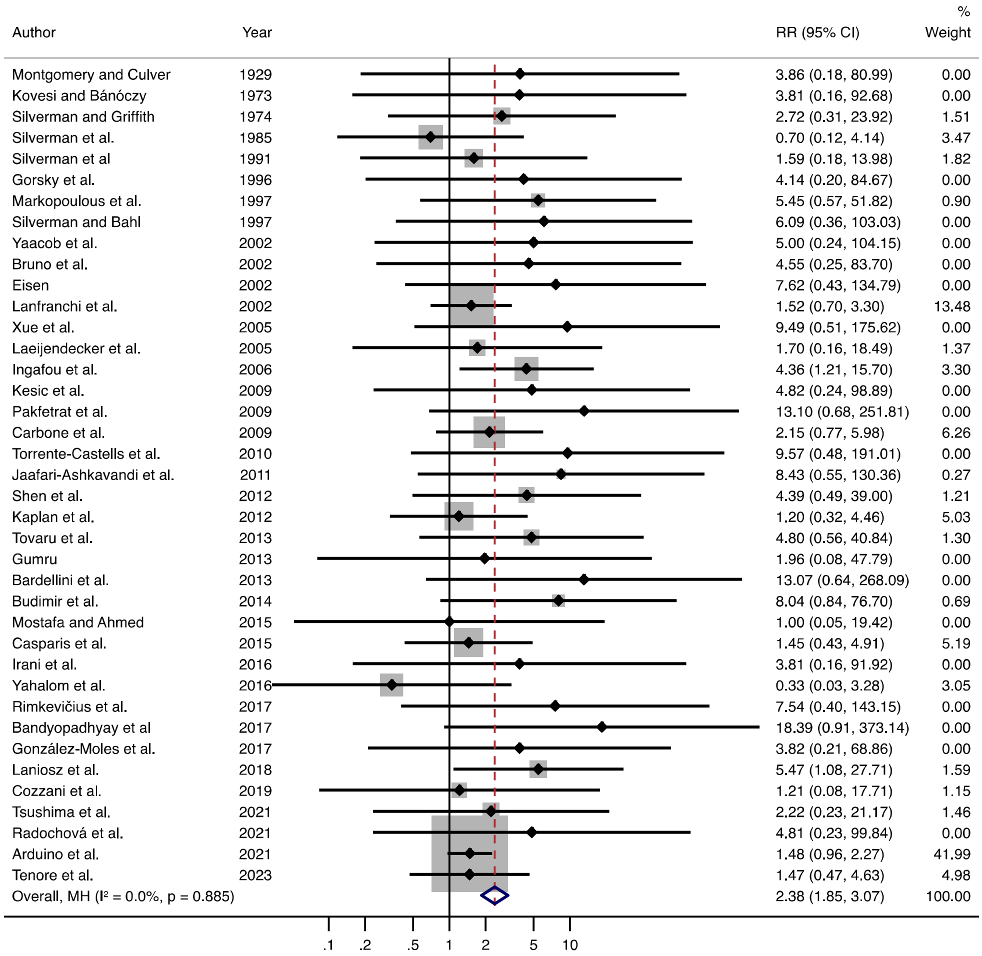

6. Tobacco and malignant transformation

Figure S7. Forest plot graphically representing the prognostic meta-analysis of the risk of oral cancer development in smokers patients with OLP (vs. non-smokers). Relative risk (RR) and 95% confidence intervals (CI) were used as effect size metric.

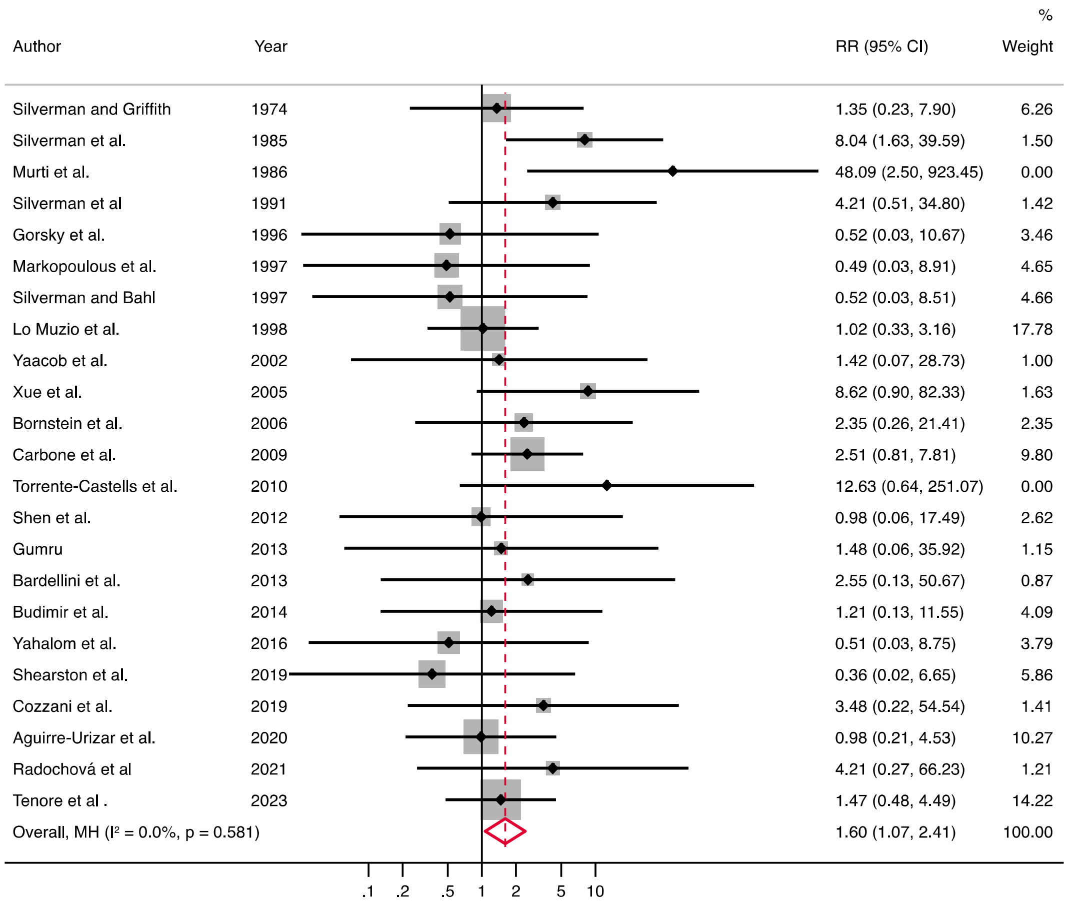

7. Alcohol and malignant transformation

Figure S8. Forest plot graphically representing the prognostic meta-analysis of the risk

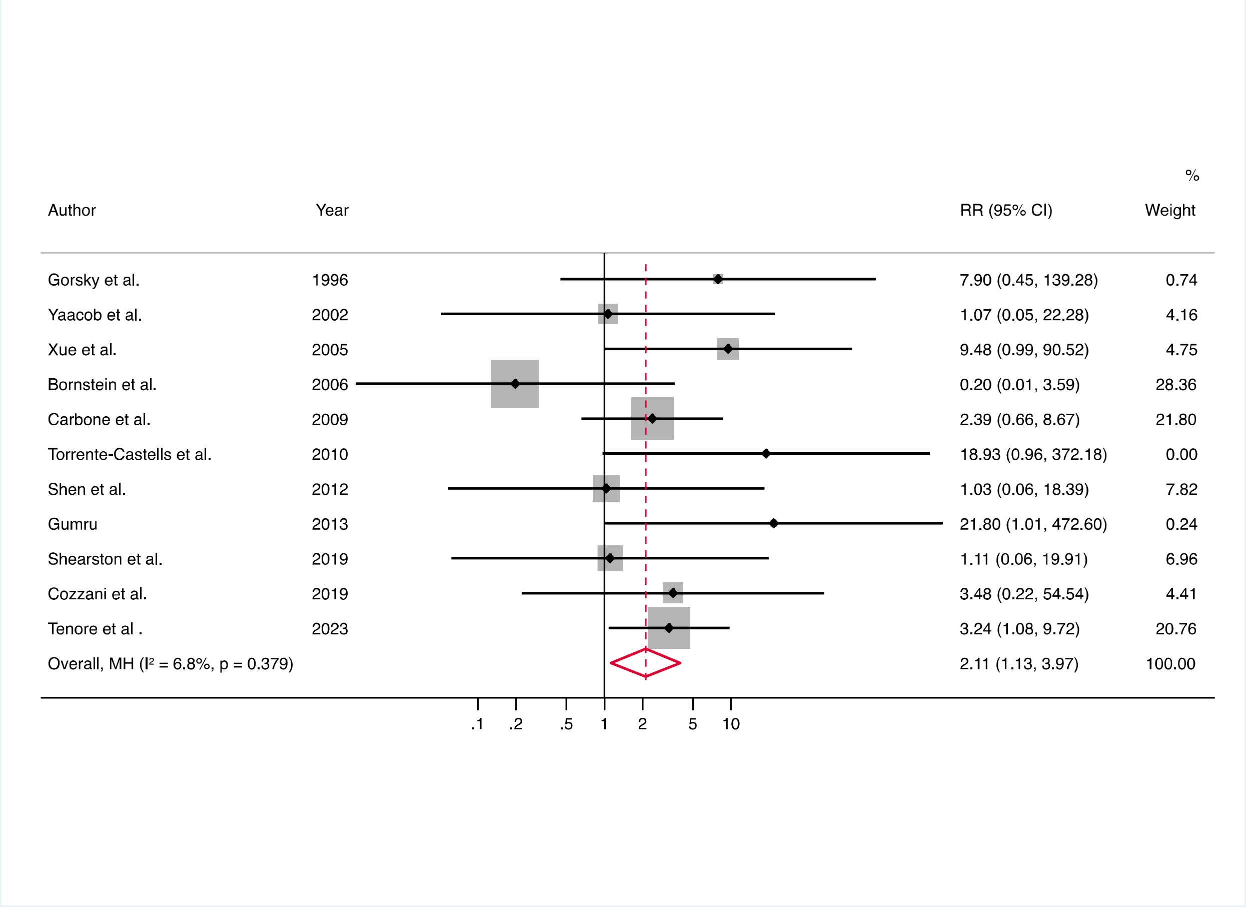

8. Hepatitis C virus and malignant transformation

Figure S9. Forest plot graphically representing the prognostic meta-analysis of the risk

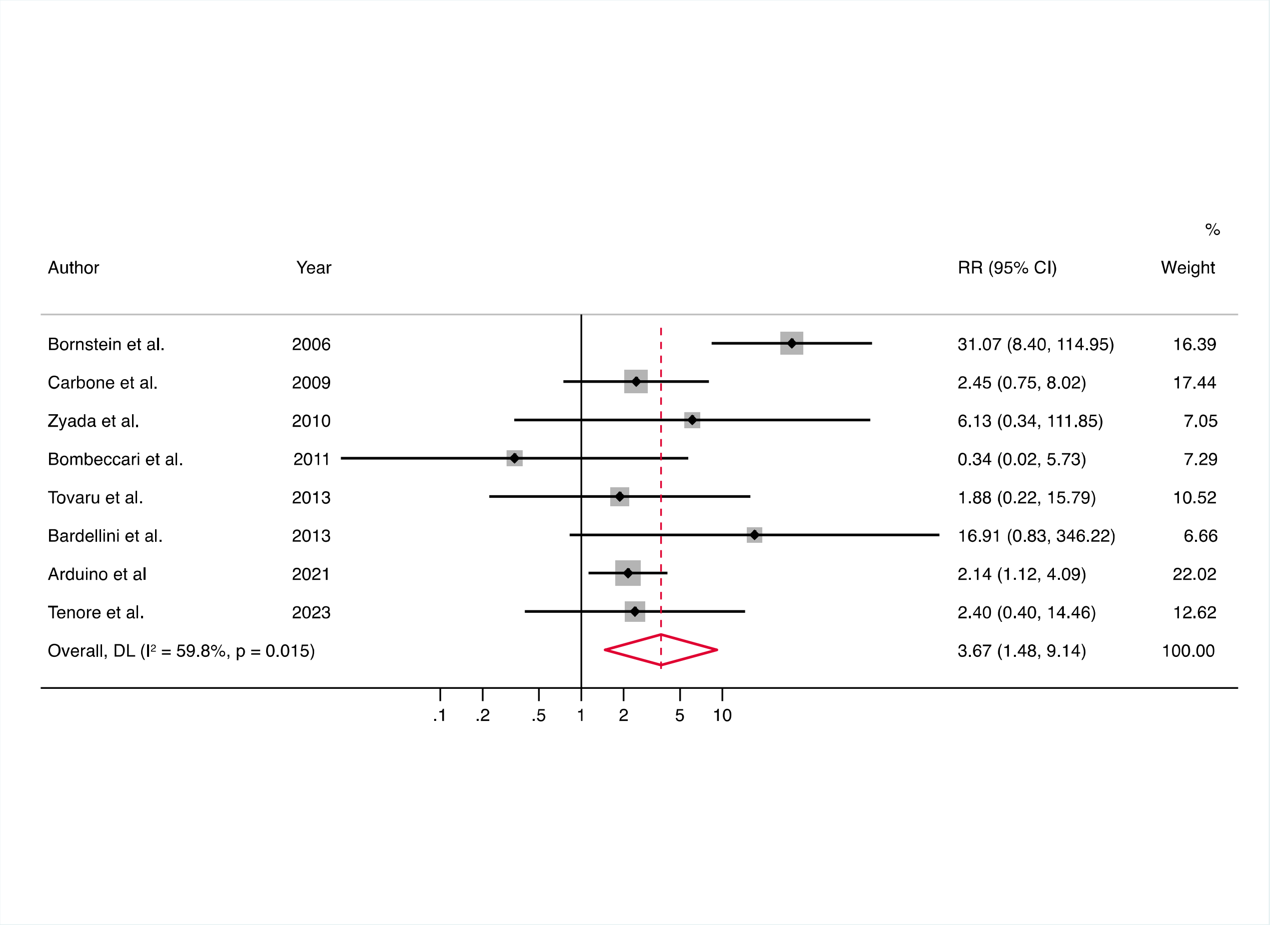

9. Localization and malignant transformation

Figure S10. Forest plot graphically representing the prognostic meta-analysis of the risk of oral cancer development in OLP lesions localized on the tongue in comparison to other sites in the oral cavity. Relative risk (RR) and 95% confidence intervals (CI) were used as effect size metric.

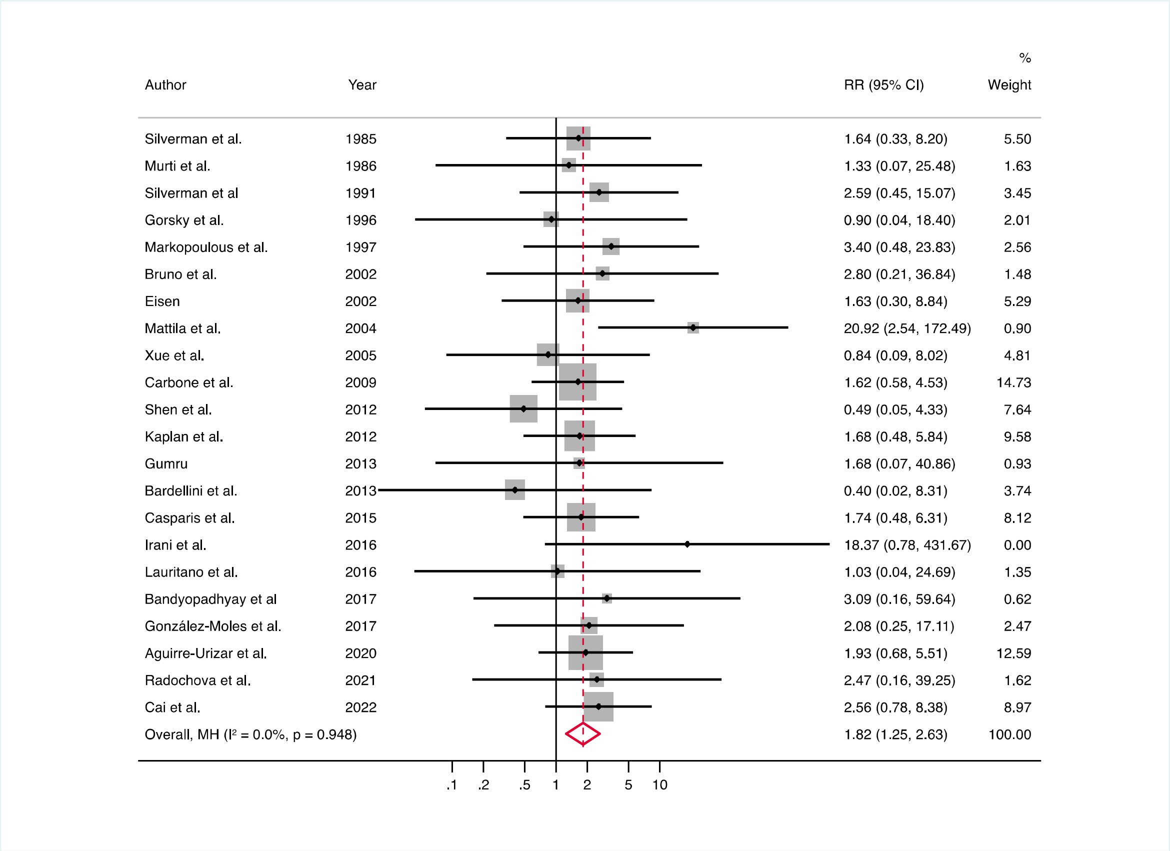

10. Sensitivity analysis on methodological quality

Figure S11. Forest plot graphically representing the sensitivity analysis on methodological quality. The subsets of primary-level studies with the highest methodological quality were included in this meta-analysis of the malignant transformation proportion (expressed as percentage) stratified by oral lichen planus and oral lichenoid lesions. Pooled proportions and 95% confidence intervals (CI) were used as effect size metric.

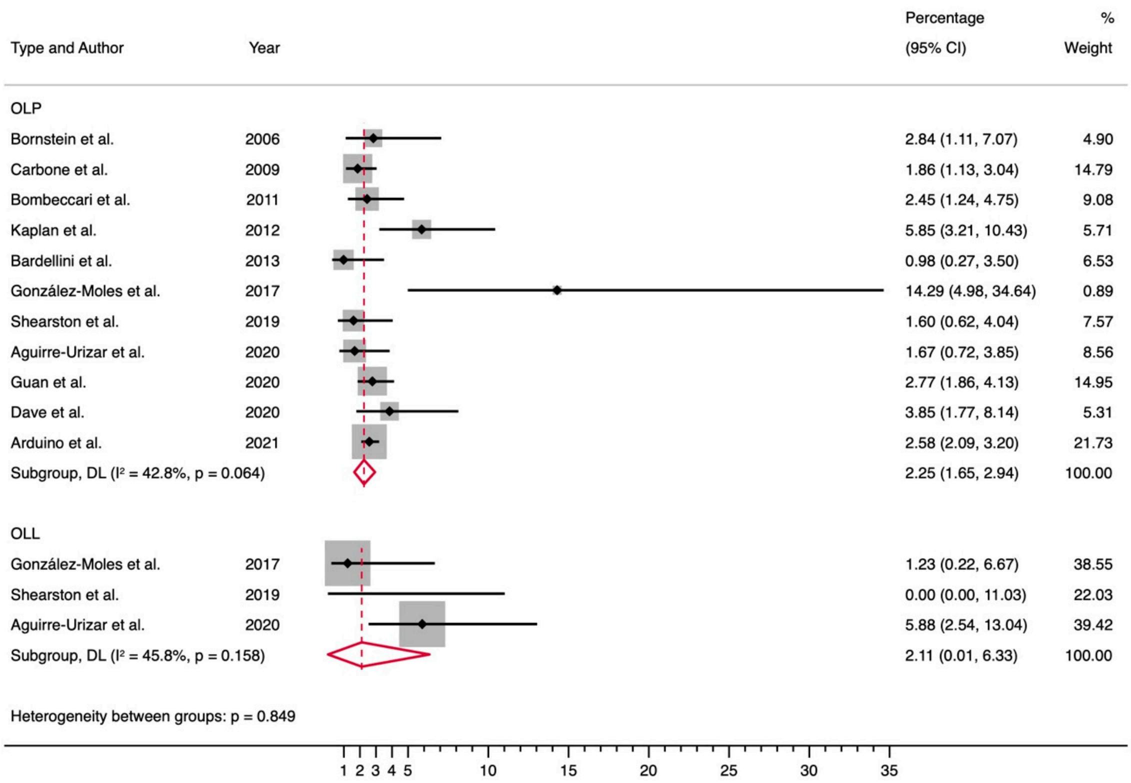

11. Analysis of small-study effects.

Figure S12. A funnel plot of estimated transformed proportions against their standard errors, graphically representing the analysis of "small-study" effects on the oral lichen planus malignant transformation ratio.

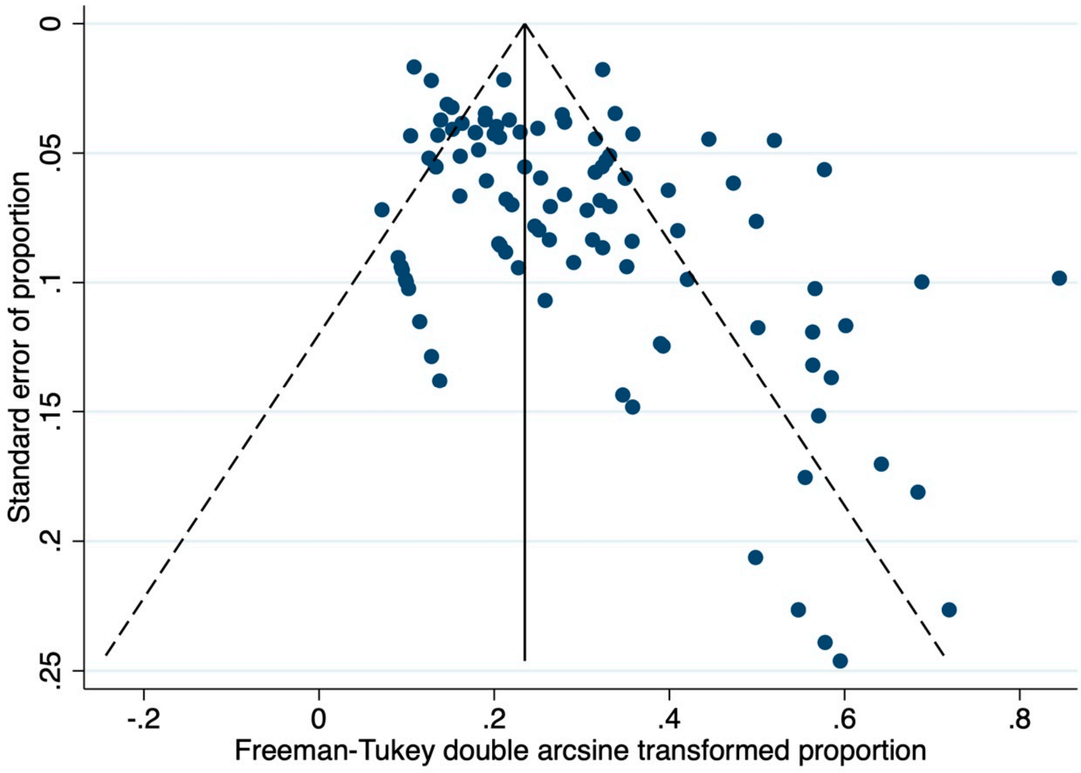

The black vertical line corresponds to the pooled estimated transformed prevalence. The two diagonal intermittent lines represent the pseudo-95% confidence interval. The blue circles represent the published studies.
